# Aedes aegypti Odorant Binding Protein 22 selectively binds fatty acids through a conformational change in its C-terminal tail

**DOI:** 10.1038/s41598-020-60242-9

**Published:** 2020-02-24

**Authors:** Jing Wang, Emma J. Murphy, Jay C. Nix, David N. M. Jones

**Affiliations:** 1 Dept. of Pharmacology, University of Colorado School of Medicine, Anschutz Medical Campus, 12801 East 17th Ave, Aurora, CO 80045 USA; 2 Program in Structural Biology and Biochemistry, University of Colorado School of Medicine, Anschutz Medical Campus, 12801 East 17th Ave, Aurora, CO 80045 USA; 3 Molecular Biology Consortium, Beamline 4.2.2, Advanced Light Source, Lawrence Berkeley National Laboratory, Berkeley, California USA; 4 Present Address: Alzheimer’s Research UK Oxford Drug Discovery Institute, NDM Research Building, University of Oxford Old Road Campus, Roosevelt Drive, Oxford, OX3 7FZ USA

**Keywords:** X-ray crystallography, Solution-state NMR, Carrier proteins, Fatty acids

## Abstract

*Aedes aegypti* is the primary vector for transmission of Dengue, Zika and chikungunya viruses. Previously it was shown that Dengue virus infection of the mosquito led to an in increased expression of the odorant binding protein 22 (AeOBP22) within the mosquito salivary gland and that siRNA mediated knockdown of AeOBP22 led to reduced mosquito feeding behaviors. Insect OBPs are implicated in the perception, storage and transport of chemosensory signaling molecules including air-borne odorants and pheromones. AeOBP22 is unusual as it is additionally expressed in multiple tissues, including the antenna, the male reproductive glands and is transferred to females during reproduction, indicating multiple roles in the mosquito life cycle. However, it is unclear what role it plays in these tissues and what ligands it interacts with. Here we present solution and X-ray crystallographic studies that indicate a potential role of AeOBP22 binding to fatty acids, and that the specificity for longer chain fatty acids is regulated by a conformational change in the C-terminal tail that leads to creation of an enlarged binding cavity that enhances binding affinity. This study sheds light onto the native ligands for AeOBP22 and provides insight into its potential functions in different tissues.

## Introduction

A critical step in disease transmission by hematophagous mosquitoes is the location of a human host for a blood meal by the female mosquito. Host location and selection of biting sites is driven by the perception of chemosensory stimuli that requires the interplay of a number of factors including chemosensory receptors and odorant binding proteins (OBPs)^1,2^. *Ae. aegypti* OBP22 (AeOBP22) is a member of the OBP family of proteins that has been directly implicated in regulating these feeding behaviors^3^. AeOBP22 is unusual in that it is expressed in multiple chemosensory tissues, including in the antenna, the proboscis of the females, the thoracic spiracles^4^, in the male reproductive glands where it is transferred to the females during mating^4,5^, and in the salivary glands^3,6,7^. Surprisingly, it was discovered that, in combination with other chemosensory genes, its expression in the salivary glands is up regulated in response to Dengue virus (DENV) infection and that knockdown of AeOBP22 using dsRNA approaches led to reduced blood feeding behaviors^3^

OBPs were first identified through their role as pheromone binding proteins (PBPs), which are components of the chemosensory apparatus that are secreted into the lymph fluid surrounding the neuronal dendrites of the chemosensory sensilla^8^. PBPs are a subgroup of OBPs that bind preferentially to pheromones. OBPs are essential for many aspects of chemosensory signal transduction^9,10^. In the lymph it is proposed that OBPs function to transport hydrophobic ligands across the aqueous lymph fluid and deliver them to chemosensory receptors^1,2,11,12^. Many studies have provided support for this hypothesis, particularly for perception of pheromonal compounds^10,13–17^. However, other studies have suggested that lymph fluid is in fact an emulsion of fatty acids and that these can enhance dispersion of pheromones into the lymph and promote interactions with the OBP^18^. Additional evidence suggests that OBPs may rather function to sequester ligands to regulate the gain/sensitivity of the chemosensory response^19,20^.

Increasingly it is apparent that OBPs are also expressed in outside of the primary chemosensory tissues including in hemolymph^21^, reproductive tissue^4,5,22–24^, as components of mosquito eggshells^23,25–27^, and as secreted components of the salivary glands of multiple insects^28,29^. In particular it has been proposed that the D7 family of OBP related proteins in mosquito saliva function to limit inflammation^30–32^, and blood clotting through their ability to sequester pro-inflammatory signals including biogenic amines and cysteinyl-leukotrienes^33^. The expression of AeOBP22 in the antenna and proboscis clearly implicates it in regulating responses to host-derived odors that emanate from skin and/or sweat that drive blood feeding behaviors, while its expression in the salivary gland suggests the potential for it to be transferred to the human host during a blood meal. It is well established that components of salivary gland extracts (SGEs) dramatically impact blood feeding behaviors and viral infectivity^33–36^. In order to better understand the potential roles of AeOBP22, we have undertaken a structural and biophysical characterization of the protein with the aim of understanding its ligand binding properties. These studies suggest that AeOBP22 may have evolved to bind to a range of fatty acids and that binding selectivity for longer chain fatty acids (>12 carbon atoms) is achieved through a conformational change in the C-terminal tail that leads to the formation of an expanded ligand binding pocket.

## Results

### NMR spectroscopy identifies long chain fatty acids as ligands for AeOBP22

**Figure 1:**
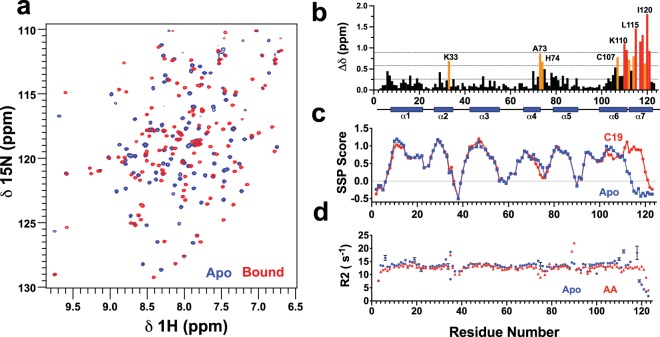
Conformational changes on binding of fatty acids to AeOBP22 **(a)** Region of the ^1^H-^15^H HSQC of apo-AeOBP22 (blue) and bound to nonadecanoic acid (red). **(b)** Plot of normalized chemical shift changes between apo-AeOBP22 and bound to C19 fatty acid. Significant chemical shift changes are color coded as greater than 1 s.d. above the mean (orange) and 2 s.d. above the mean (red). Horizontal dashed lines indicate the position of the mean, +1.sd. and +2 s.d. from bottom to top. The location of the α-helical regions is shown as blue cylinders below. **(c)** Plot of secondary structure propensity scores (SSP)^41^ for apo AeOBP22 (blue) and the complex with C19 (red). **(d)** Plot of NMR ^15^N R_2_ relaxation rates for apo (blue) and bound to arachidonic acid (red) recorded at a ^1^H frequency of 900 MHz. Error bars are shown in black.

We used NMR spectroscopy to screen compounds from human sweat and skin, repellents, fatty acids and bioactive lipids for binding to AeOBP22. We prepared purified, delipidated protein^37–39^ by extensively washing isolated inclusion bodies using buffer containing 1 M urea followed by refolding^40^. Retrospectively, we determined that “non-delipidated” samples contain a mixture of the apo-state of the protein and the complex formed with palmitic acid (16 carbons) (Supplementary Fig. 1). The ^1^H-^15^N HSQC spectrum of the fully delipidated sample (Fig. 1a) shows 119 of the expected 120 peaks for the backbone amides. In NMR screening, we consistently found that longer chain fatty acids (C16–C20 carbons) produce large changes in the appearance of the NMR spectrum at low concentrations **(**Fig. 1a). Other compounds can produce the same magnitude of NMR chemical shift changes, notably geraniol, citronellol and benzaldehyde (Supplementary Fig. 2**)**. However, this only occurred at high ligand concentrations (typically> 1 mM) and binding is weak as exemplified by the concentration dependence of the chemical shift changes. In contrast, fatty acids bind with high affinity as evidenced by the presence of peaks from the free and the bound states present in slow exchange on the NMR timescale when the ligand is present in sub-stoichiometric quantities (Supplementary Fig. 3). From the NMR chemical shift assignments of the apo-protein and the complexes with nonadecanoic (C19) acid and arachidonic acid (AA)^40^ we determined that fatty acid binding has the largest impact on residues 106–121 in the C-terminus (Fig. 1b). Further calculations of the secondary structural propensities (SSPs) from the chemical shift data^41^ predict that the C-terminal tail adopts an extended conformation in the apo-state and adopts an α-helical conformation when bound to longer chain fatty acids (Fig. 1c)^40^.

### Crystal structures of AeOBP22 complexes

Next we used X-ray crystallography to better define the conformational changes that occur on binding fatty acids and we determined structures in three different crystal forms. Initial crystals formed in the P3_1_21 space group, and the structure was solved using single wavelength anomalous dispersion (SAD) of a tantalum bromide (Ta_6_Br_12_) soaked crystal refined at 2.6 Å resolution. A native data set collected on the same crystal was refined at a resolution of 1.9 Å to an R/R_Free_ of 18.3/20.1% (Supplementary Fig. 4a). In this crystal form the protein forms a domain swapped dimer, with residues 116–122 of one molecule forming an anti-parallel β-sheet with residues 37–41 in a symmetry related molecule **(**Supplementary Fig. 4b).

**Figure 2:**
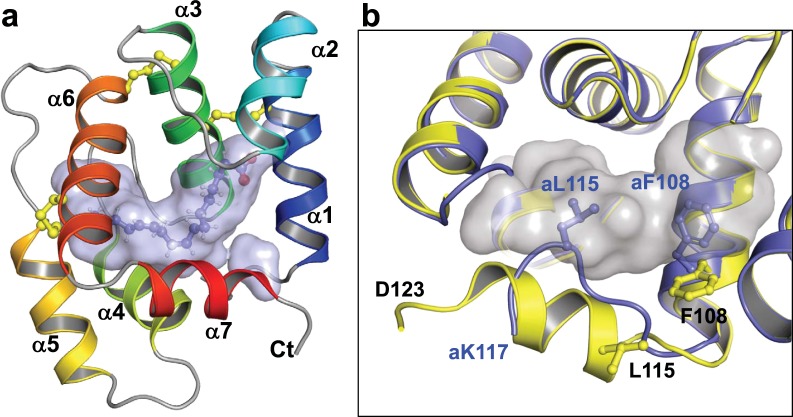
Structure of AeOBP22. **(a)** Ribbon diagram of the AeOBP22-linoleic acid complex solved by X-ray crystallography at 1.85 Å. Helices are color coded from blue to red, N to C terminus. The binding pocket is shown as a surface representation and linoleic acid is shown as sticks. **(b)** Comparison of the C-terminal tail in the linoleic acid complex (yellow) and in the apo-state (blue). In apo AeOBP22, L115 and F108 insert into the pocket (shown in grey) to occlude binding of larger ligands. Reside numbers for the apo-state are preceded with an “a”. Residues 118–123 of the apo-state are not observable in the crystal.

**Figure 3:**
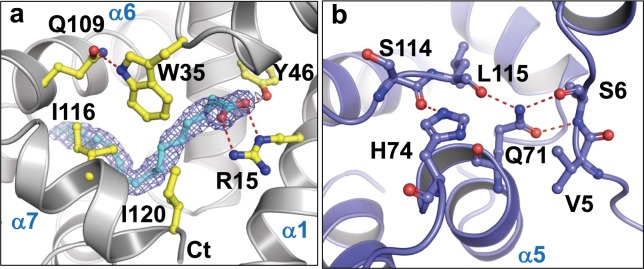
Critical interactions that stabilize the in the bound and apo-states of AeOBP22 **(a)** Electron density for linoleic acid in a 2Fo-Fc omit map contoured at 1σ with the fatty acid modelled in cyan. Y46 and R15 make specific H-bonds with the fatty acid. Positioning of the head group is reinforced by interactions with I120, I116, and W35, which H-bonds to Q109 in helix-6. **(b)** In apo-AeOBP22, the position of the C-terminal tail is stabilized by H-bonds between S114 and L115 with the side chains of H74 in the α4-α5 loop and Q71 in helix-4, which in turn forms hydrogen bonds with S6 in the N-terminal region.

The second crystal form was solved in the P3_1_ space group and contains nine molecules in the asymmetric unit arranged in a pseudo three-fold arrangement of “trimers” consisting of domain swapped dimer and a separately packed monomer (Supplementary Fig. 5). The dimers are identical to those observed in crystal form 1 and superimpose with an average pairwise RMSD of 0.47 Å. The individual monomers superimpose to each other with an average pairwise RMSD of ~0.2 Å. Ligands are observed bound at the center of each monomer (Fig. 2a and 3a) with residues 112–121 forming an α-helix that forms one edge of the ligand-binding pocket (Fig. 2b). No ligand is observed in the dimeric components. In this form we solved the structures with palmitic acid (C16:0), palmitoleic acid (C16:1), and eicosanoic acid (C20) complexes.

The third crystal form grows in the presence of cadmium and/or cobalt ions in the C121 space group and contains only monomers. Structures of the apo-state and the complexes formed with linoleic acid (C18:2) and AA (C20:4) were solved using cobalt SAD methods and refined to a resolution of 1.85 Å. Data collection and refinement statistics for deposited structures are given in Supplementary Table 1.

### AeOBP22 is monomeric in solution

Previous studies have proposed that dimerization of OBPs may regulate ligand binding and transport^19,42–47^. Therefore, we used measurements of ^15^N NMR relaxation rates*, R*_1_ and *R*_2_, to determine if AeOBP22 forms dimers or higher order complexes. For a globular protein there is a direct correlation between the average R_2_/R_1_ ratio and the molecular weight^48–51^. For AeOBP22 the predicted molecular weights from multiple measurements of the R_2_/R_1_ ratios in both apo and fatty acid bound states (Supplementary Fig. 6**)** were in the range 14.1–15.2 KDa, in agreement with the expected molecular weight of 14.3 KDa, indicating that the protein exists as monomers in the absence of other binding partners. We saw no evidence of any significant dimerization over long periods even at the high concentrations used for NMR chemical shift assignments (600–700 μM). Therefore, we conclude that the domain swapped dimers are an artifact of the crystallization process, and further discussions below are confined to the structures of the monomeric forms.

A complete analysis of the relaxation data using Relax^52–54^ (Supplementary Fig. 7) revealed slightly elevated exchange contributions to the relaxation rates for residues 111–118 suggesting that this region of the protein may undergo conformational exchange on slower time scales, and this may be important to allow access of fatty acids to the ligand binding pocket. This analysis also confirms that residues 74–78 exhibit increased conformational flexibility in the bound state compared to the apo-state.

### Description of structure

The monomeric forms of AeOBP22 are similar to other classical OBPs which consist of six α-helices stabilized by three disulfide bridges surrounding a hydrophobic pocket (Fig. 2a). In the bound state, AeOBP22 is unusual in that it contains a seventh C-terminal α-helix that forms one edge of the ligand-binding pocket. The ligand-bound monomers from all our structures superimpose well with a pairwise RMSD of 0.40 Å **(**Supplementary Fig. 8). Small differences are seen in the position of helix-7, which rotates out slightly from the binding pocket in the presence of larger fatty acids such that the Cα at Ile120 is approximately 2 Å displaced from its position with shorter chain fatty acids. Additionally, we see variations in the position of the α4-α5 loop (residues 73–78), which may be a result of crystal packing interactions.

The ligand-binding pocket is in the form of a long tunnel approximately 20 Å in length and occupies 144 ± 10 Å^3^ calculated using CASTp 3.0^55^, and the alkyl chain of the ligand contacts hydrophobic residues that line the pocket (Supplementary Fig. 9). At the opening of the pocket there is an electrostatic patch formed by Arg15, Lys33 and Lys117 (Supplementary Fig. 10). Arg15 in combination with Tyr46 make multiple specific H-bonds to the carboxyl group of the fatty acid (Fig. 3a**)** and together define the requirement for a negatively charged group at this position. The fatty acid head group also contacts Trp35, which in turn hydrogen bonds to Gln109. All these residues are highly sensitive to ligand binding in NMR experiments.

At the distal end of the pocket from Arg15, three highly ordered water molecules hydrogen bond to Trp100, Ala101, Gly104, Cys88 and Val89. Analogous water molecules are observed in the binding pocket of *Ae. aegypti* Juvenile Hormone Binding Protein (AeJHBP)^56^. This suggests that AeOBP22 may accommodate ligands with polar groups at this position. To test this, we examined the interactions with 16-hydroxy-hexadecanoic acid (C16-OH) and observed large NMR chemical shift changes relative to the apo-protein that are comparable to the changes observed with C16 (not shown). We observed additional changes for residues 86–92 that are consistent with the interaction of the C16-hydroxyl group at this position (Supplementary Fig. 11**)**. However, residues throughout helices 6 and 7 (103–121) show line broadening and reduced intensity indicative of increased conformational averaging. Additionally, we found that C16-OH does not compete for binding of a fluorescent reporter in ligand binding assays (below) and so we conclude that distal polar groups appear to be unfavorable for binding and destabilize the conformation of the AeOBP22 complex, suggesting that the role of the buried water molecules is more likely to be as a structural component.

### Structure of the apo-state of AeOBP222

The structure of apo-AeOBP22 confirms that residues 118–123 are disordered and 112–117 adopt an extended structure compared to the α-helix observed in the bound state (Fig. 2b and Supplementary Fig. 8b). In this extended conformation, Leu115 and Phe108 insert into the core of the protein and restrict the size of the binding pocket (Fig. 2b). This extended structure is stabilized by the formation of hydrogen bonds between Ser114 and Leu115, with His74 and Gln71 in the α4-α5 loop respectively (Fig. 3b). In turn, Gln71 hydrogen bonds with Ser6 in the N-terminus. This network of interactions links conformational changes in the C-terminus to the N-terminus through the α4-α5 loop. This explains our NMR relaxation data (Fig. 1d and Supplementary Fig. 6 and 7), which shows increased conformational mobility of the α4-α5 loop on binding fatty acids because these interactions are disrupted.

### NMR Solution Structure of AeOBP22 with Arachidonic Acid

**Figure 4:**
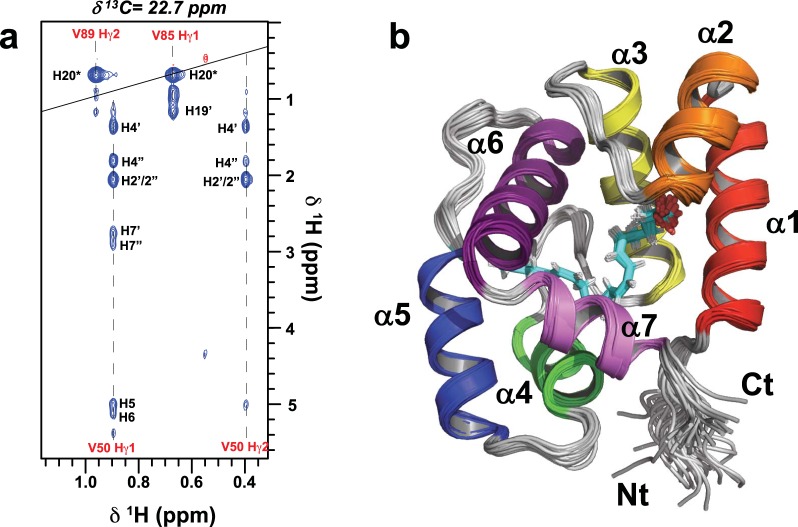
NMR Structure of the AeOBP22-AA Complex **(a)** A slice through a ^12^C-edited/^13^C-filtered intermolecular NOESY spectrum at δ^13^C = 22.7 ppm showing NOEs between resonances from the protein (labeled in red) and arachidonic acid (black labels). For the lipid the hydrogens are numbered according to the attached carbon in the alkyl chain. **(b)** Superposition of the 30 lowest energy structures from the final iteration of NMR calculations (RMSD = 0.32 Å for res 7–121), helices 1–7 are color coded from red-violet. The arachidonic acid is shown in cyan.

In parallel with our crystallographic studies we determined the NMR solution structure of the AeOBP22-AA complex. AA was chosen because the vinylic protons of this fatty acid each have a unique chemical shift and are in a region of the spectrum (4.6–5.6 ppm in ^1^H) that has minimal overlap with resonances from the protein^40^. This increased chemical shift dispersion greatly facilitated the assignment of intermolecular NOEs between the protein and the ligand (Fig. 4a). The ensemble of 30 lowest energy structures from the final round of calculations superimpose with a backbone RMSD of 0.32 Å from the mean structure, while the carbon atoms in the ligand superimpose with an overall RMSD of 0.31 Å (Fig. 4b**)**. The list of structural restraints and refinement statistics are given in Supplementary Table 2.

The NMR structure of the AeOBP22-AA complex superimposes with the crystal structure with an RMSD of 0.67 Å for the backbone atoms of residues 7–120 and confirms that the monomeric structure observed in the crystal is maintained in solution. The biggest differences between the structure are in the conformation of the loop between helices 4 and 5, which are involved in crystal packing, but which are dynamic in solution. The overall structure of the arachidonic acid ligand is similar to the structure observed in the crystal. However, even in the crystal there are differences in the ligand position in the two monomers in the asymmetric unit.

### Impact of Chain Length on the Conformation of the C-terminal tail

**Figure 5:**
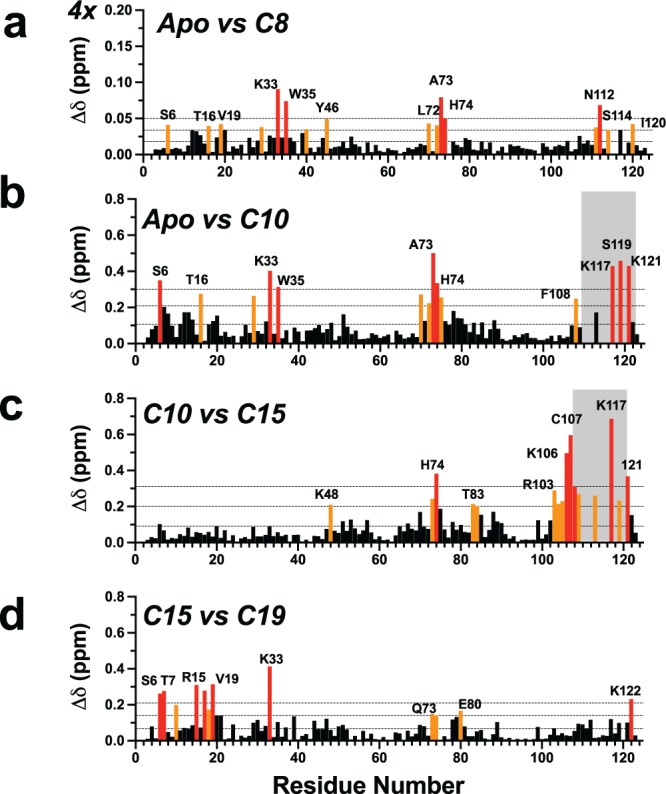
Chemical shift changes as a function of fatty acid chain length. Comparison of chemical shift differences in the ^1^H-^15^N HSQC spectra recorded at 600 MHz for **(a)** apo compared to octanoic acid. **(b)** Apo versus C10. **(b)** C10 acid vs C15 acid and **(d)** C15 vs C19 acids. Significant chemical shift perturbations are color coded as in Fig. 1. In panel (a), the vertical scale has been expanded by four compared to the other panels. For C10–C14 conformational averaging in the C-terminal tail limited the assignments for a number of residues in this region (shaded in grey in b and c). In all cases the protein was at ~100 μM and the fatty acid was present at a concentration of 200 μM in sodium phosphate at pH 6.5.

In the apo-AeOBP22 structure we observed a small binding pocket located between Arg15 and Leu115 that could potentially accommodate short chain fatty acids (up to C6). When we examined the binding of fatty acids containing up to 8 carbons by NMR, we observed chain length dependent chemical shift changes for residues in the N-terminal half of the protein (Fig. 5a and Supplementary Fig. 12a**)**, that are consistent with binding of the carboxylate group to the basic residues in the vicinity of Arg15, Lys33 and Trp35 **(**Supplementary Fig. 12b**)**. To ensure that addition of free fatty acids did not result in changes in pH that could perturb the NMR spectrum, we examined chemical shift changes caused by pH alone (Supplementary Fig. 12b**)**. We observed that the patterns and magnitude of chemical shift changes caused by short chain fatty acids are distinct from those caused by changes in pH alone. It was surprising that C8 still binds with relatively little impact on the overall structure suggesting a significant plasticity in the binding pocket. However, the exact mode of binding of short chain fatty acids remains to be definitively established.

In contrast to short chain fatty acids, large chemical shift perturbations are observed throughout the protein in the presence of decanoic acid (C10) (Fig. 5b). After reassigning the spectrum, we determined that C10 binding impacts the C-terminal tail, the α4-α5 loop, the end of helix-2 (residues 33–35) and helix-1. Several residues in the C-terminal tail show increased exchange broadening and could not be assigned (shaded in grey in Fig. 5b,c). Increasing the alkyl chain from C10 to C15, leads to additional perturbations in the C-terminus (aa 109–121) and in the α4-α5 loop (aa 70–75). However, when the alkyl chain is further increased from C15 to C19 additional chemical-shift perturbations localize predominantly to residues 6–21 in α-helix 1 (Fig. 5d), with limited changes in the C-terminal region. There are no additional changes beyond C20, and fatty acids with more than 20 carbon atoms show a dramatic reduction in their ability to bind (not shown). We conclude that fatty acids with at least 10 carbon atoms are required to stimulate a conformational change in the C-terminus, and this is maximally induced with a chain length of 15–16 carbon atoms. Secondly, a conformational shift in helix-1 is required to accommodate binding of longer chain fatty acids C17–C20. This adaptation differs to that observed in the crystal, where helix-7 shifts in response to longer chain fatty acid. We attribute this to the crystal packing contacts formed by helix-1 which restricts its ability to move. In contrast, helix-7 makes few crystal contacts and has greater ability to adapt its conformation to accommodate different ligands.

### Fatty acid chain length and degree of unsaturation impact the binding affinity to AeOBP22

**Figure 6:**
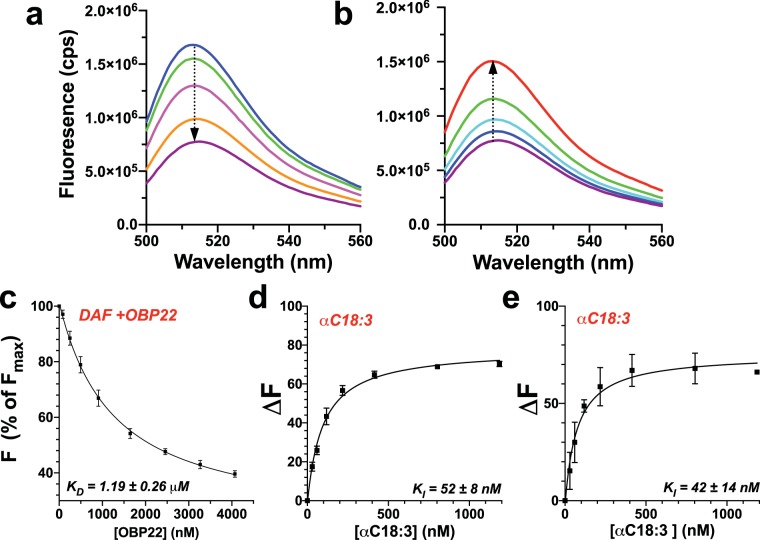
Long chain fatty acids bind with high affinity to AeOBP22. **(a)** Florescence emission spectra of DAF (blue) excited at 490 nm, with increasing amounts of AeOBP22 (arrow) shows concentration dependent quenching. **(b)** Titration of the final sample in a, with increasing linoleic acid (arrow) shows that the fatty acid competes for binding of DAF and recovery of initial fluorescence (red). **(c)** Determination of binding constant for AeOBP22 and DAF from 7 replicates of experiments shown in panel (a). DAF concentration was 84 nM. Plot of the intensity at the florescence maximum (513 nm) presented as change from initial fluorescence. Data were fit according to Eq. 2. **(d)** Determination of the binding constant of α-linoleic acid at pH 8.0 from multiple replicates (n = 3) of experiments shown in panel (b) by fit of the raw intensity using Eqs. 2 and 3 using a K_D_ for DAF of 1.191 μM. The DAF concentration was fixed at 100 nM and the protein at 1000 nM. Data are presented as normalized recovery of the quenched fluorescence for ease of viewing. **(e)** The same experiment as in (d) but recorded at pH 5 (n = 2). Intrinsic DAF fluorescence is significantly quenched at pH 5.0 leading to increased error in measured points.

Next, we asked how the chain length and presence of unsaturation impacts the binding affinity for AeOBP22. Many previous studies have used 1-NPN as a fluorescent reporter to examine ligand binding to OBPs^57,58^, including for AeOBP22^4,59^. In our hands, we found that 1-NPN was unsuitable for studies of fatty acid binding as it bound with low affinity and showed non-specific interactions that confounded analysis. Further, studies with 1-NPN are often confounded by the ability of ligands to bind simultaneously and quench 1-NPN fluorescence rather than bind in a competitive manner^60^. Therefore, we investigated the use of a fluorescently labeled fatty acid derivative 5-(N-dodecanoyl)-amino-fluorescein (DAF), as a reporter for competition binding assays. DAF shows a strong fluorescence emission with a maximum at 513 nm which is quenched upon addition of AeOBP22 (~75% reduction from maximal intensity) and with a *K*_D_ of 1.19 ± 0.26 μM (n = 7) (Fig. 6a,c). Addition of fatty acids with 14 or more carbon atoms releases DAF from AeOBP22 leading to a recovery of the initial fluorescence (Fig. 6b,d). In addition, NMR spectroscopy shows that DAF produces chemical shift changes comparable to those produced by C11–C14 fatty acids **(**Supplementary Fig. 13**)**, and conformational line broadening for residues in the C-terminal region. Therefore, we conclude that the alkyl chain of DAF likely binds to the central pocket of AeOBPP2 and can displace the C-terminal tail, and that fatty acids bind competitively with DAF.

We used DAF based competition binding assays to determine binding affinities for a series of fatty acids (Table 1 and Supplementary Fig. 14). This revealed that the both chain length an unsaturation impact the binding affinity. Saturated C16 binds weakly with *K*_D_ ~690 nM, however, introduction of a single degree of unsaturation at the Δ9 position (C16:1) leads to an approximate four-fold increase in the binding affinity (K_*D*_ = 175 ± 41 nM). Increasing the chain length and the introduction of additional unsaturation enhances binding, which appears to be optimal for the fatty acids containing 18 carbon atoms, as increasing the chain length to C20 leads to a two-fold reduction in affinity. However, the extent of unsaturation also impacts the affinity, with the more rigid AA (C20:4) having a lower affinity than the monounsaturated C20:1. These results are consistent with our NMR data that shows binding of longer chain fatty acids requires a conformational change in α-helix 1 (Fig. 5d) and so this appears to be unfavorable for binding.

**Table 1:** Dissociation constants for binding of fatty acids to AeOBP22.

Ligand	K_D_ (nM)^1^
DAF	1191 ± 261^2^
C16:0	689 ± 89
C16:1	175 ± 41
C18:1	84 ± 5
C18:2	104 ± 30
αC18:3 pH 8.0	52 ± 8
pH 5.0	42 ± 14^3^
γC18:3	111 ± 27
C20:1	102 ± 16
C20:4	286 ± 29

^1^Values reported in nM and ± the standard deviation, n = 3.

^2^n = 7.

^3^n = 2.

### Changes in pH do not impact the binding of fatty acids

In apo-AeOBP22, His74 makes interations that maintain the C-terminal residues in a conformation that occludes the ligand binding site. Consequently it is possible that pH may impact the conformation of the C-terminal residues and ligand binding in a manner somewhat analogous to that observed for PBPs^61–64^. Indeed, previous studies of AeOBP22 have suggested that the helical content of the protein increases at lower pH^59^. To test this, we compared the binding affinity for α-linoleic acid at pH 6.5 and 5.0 but found no significant difference at these two pHs, 52 ± 8 nM vs 42 ± 14 nM respectively (Fig. 6d,e and Table 1). We could not use DAF below pH 5.0 because its intrinsic fluorescence is severely quenched. Therefore, we used NMR to examine the effect of pH on the protein structure and binding. For these studies, samples were prepared in sodium citrate at pH 6.5 and the pH adjusted using hydrochloric acid over the range 6.5–4.5. We found that lowering the pH resulted in chemical shift changes consistent with protonation of His74 and His70 between pH 6.5 and 5.5 (Supplementary Fig. 14b**)** and sidechain carboxyls between pH 5.5 and 4.5 (not shown). However, the cumulative chemical shift changes (~0.3 ppm) over the pH range 6.5–4.5 are 5–6 times smaller than the perturbations induced by the binding of chain fatty acids (Fig. 1), indicating that changes in pH do not induce the conformational rearrangement observed with fatty acids. To validate this, we obtained backbone chemical shift assignments (^1^H, ^15^N, ^13^Cα, ^13^Cβ) for both the apo-protein and the α-linoleic acid complex at pH 4.5 and examined differences in the secondary structural propensities and the normalized chemical shift differences between the two samples (Supplementary Fig. 15). These almost exactly matched the SSPs and patterns of chemical shift changes upon ligand binding observed for samples recorded at pH 6.5, and confirms that pH does not impact the structure of the apo-protein or the ability of AeOBP22 to bind to longer chain fatty acids at low pH.

### Comparison of AeOBP22 with known structures

**Figure 7:**
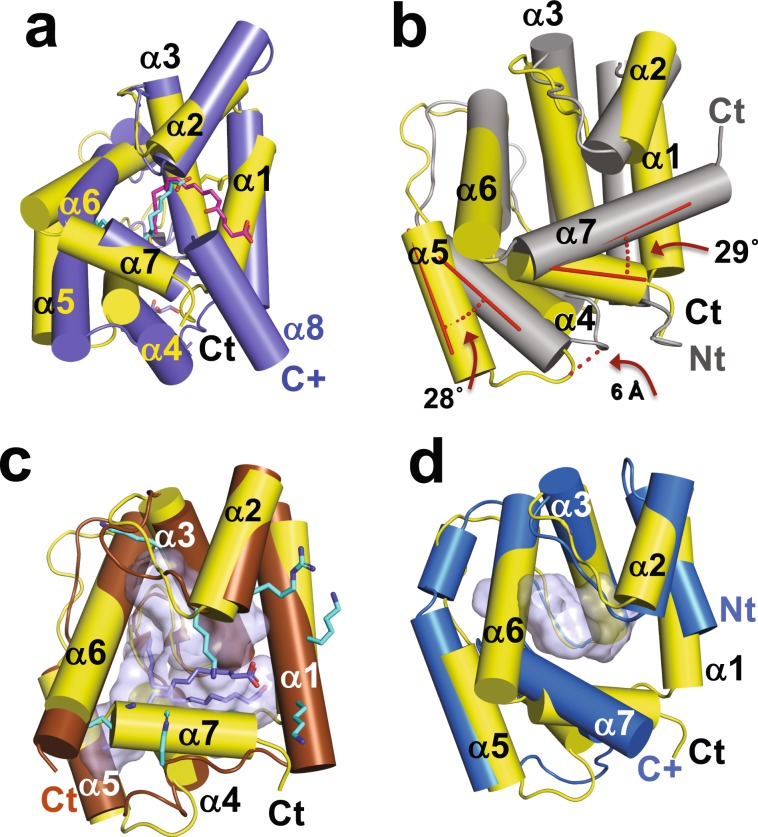
Comparison of AeOBP22 with known structures. Cylinder representation of AeOBP22 in yellow (N and C-termini labeled in black) compared with **(a)** N-terminal OBP domain of *An. stephensi* D7 (blue) bound to Leukotriene C4 magenta (PDB 3NHI)^67^. For clarity, the C-terminal domain, which continues at the position labeled C+ in blue, is not shown. The linoleic acid bound to AeOBP22 is shown in cyan. **(b)** LmigOBP1 (grey) (PDB 4PT1)^69^. The difference in the positions of helices 5 and 7 are shown in red. **(c)** *Phormia regina* OBP56a bound to multiple molecules of palmitic acid (blue) (PDB 5DIC) (Ishida *et al*. not published). The multiple lysines and arginines that surround the pocket (grey) are shown in cyan. **(d)** *An. gambia*e OBP22 (bright blue) (PDB 3L4L) **(**Zhang and Ren, not published). The C-terminus of AgOBP22 is mostly unstructured (extends from position labeled with blue C+).

The bound state of AeOBP22 contains a seventh α-helix at the C terminus that forms one edge of the ligand-binding pocket. A DALI search^65,66^ shows the highest structural similarity to the N-terminal domains of the insect D7 proteins that contain dual OBP-domains; *Ae. aegypti* juvenile hormone binding protein^56^ (AeJHBP, PDB-ID 5V13) with a DALI Z-score of 15.4, *An. stephensi* D7^67^ (AsteD7, PDB 3NHT, Dali Z = 12.8), and A*n. gambiae* D7-Leukotriene E4 complex (AgamD7, PDB 3DZT, Dali Z = 12.2)^31^. The seventh helix of AeOBP22 is in a remarkably similar position to the seventh helix of these D7 proteins (Fig. 7a, only the N-terminal domain of AsteD7 is shown). However, in the D7 proteins the binding pocket is not as deep, and the ligand (magenta in Fig. 7a) extends out of the binding pocket and contacts residues in the C-terminal domain (not shown).

There are three structures of single domain OBPs that have an additional seventh C-terminal helix; *An gambiae* OBP7 (PDB 3R1O)^68^, *Locusta migratoria* OBP1 (LmigOBP1, PDB 4PT1)^69^ and the Mediterranean fruit fly, *Ceratitis capitate* OBP22 (PDB 6NHE)^70^. Of these, AeOBP22 is most similar to LmigOBP1 (Dali Z = 12.1) (Fig. 7b**)**, however, helix 7 is significantly longer in LmigOPB1 and the angle between helices 6 and 7 differs by ~30° (red in Fig. 7b). Additionally, in AeOBP22 helices 4 and 5 are rotated out from the main body of the protein by ~28°. As a consequence, AeOBP22 has a longer, more extended pocket compared to that in LmigOBP1 (170 Å^3^).

AeOBP22 additionally shows structural conservation with the blowfly, *Phormia regina*, OBP56a (PregOBP56a, PDB 5DIC, Dali Z = 13.7) (Ishida *et al*. not published), which is proposed to transport fatty acids for feeding^71^. Helices 1 through 6 of PregOBP56a superimpose with AeOBP22 with an RMSD of 1.2 Å (Fig. 7c). However, PregOBP56a does not have a seventh helix. Instead, the N-terminal residues 1–5 occupy a similar position to the C-terminus of AeOBP22. The binding pocket of PregOBP56a is significantly larger (~530 Å^3^) and is lined by multiple lysine residues. This allows it to accommodate multiple ligands in a non-specific manner (Fig. 7c**)**. This contrasts with AeOBP22, which has a hydrophobic pocket with Arg15 and Tyr46 positioned to make specific hydrogen bonds with carboxylic acid containing ligands (Fig. 3a).

AeOBP22 also has structural homology to *An. gambiae* OBP22 (AgamOBP22, PDB 3L4L, Dali Z = 12.5) (Zhang and Ren, not published) (Fig. 7d). AgamOBP22 differs in having a significantly shorter N-terminal region but simultaneously a much longer C-terminus, which is only partially observable in the deposited structures. This C-terminal region also forms a seventh α-helix, however the angle formed with helix-6 is more obtuse, ~116° compared to ~75°, seen in AeOBP22 and the binding pocket is relatively shallow and smaller (123 Å^3^) than that of AeOBP22.

## Discussion

It is increasingly apparent that insect OBPs have diverse roles that extend beyond those of chemosensory signaling^72^. AeOBP22 is a prime example of this as it is expressed in multiple tissues^3–7^. Other OBPs expressed in multiple tissues include two related *Helicoverpa* spp., where it is proposed that HeOBP10 functions in both the delivery of an oviposition deterrent to fertilized eggs, and in the subsequent detection of that deterrent in the antenna^23^. Understanding the native ligands that interact with OBPs can provide insights into the underlying biology. Our results provide evidence that fatty acids are likely natural ligands for AeOBP22 and that the binding of long-chain fatty acids (C15-C20) is enhanced by a conformational change in the C-terminal tail that is critical to generate a high affinity binding site. Previously we showed that burial of a single methylene group from a ligand contributes ~1 Kcal mol^−1^ to the overall stability of the Drosophila OBP LUSH^73^ and so burial of a long alkyl chain can contribute significant binding energy leading to high affinity interactions.

Conformational changes associated with ligand binding have been demonstrated for multiple OBPs and PBPs^37–39,61,63,64,74–78^. These can stabilize the structure of the protein^17,45,73,79^, accommodate different ligands^68,74,75,80,81^ or displace ligands^37,61,64,76–78,82,83^. In *Antheraea polyphemus* PBP1, in the absence of ligand the C-terminal tail forms a long α-helix and occupies the binding site^37^. It functions to both displace ligands at lower pH but also contributes to high affinity ligand binding at higher pH^84^, even though it is displaced from the binding site, and so the mechanism for how it does this remains uncertain. The conformational change observed for AeOBP22 is more similar to the coil-to-helix transition observed for binding of norepinephrine to the C-terminal domain of *Ae. aegypti* D7 that results in capping of the ligand-binding pocket^31^. In the absence of ligand this C-terminal tail is highly dynamic. In contrast, the C-terminal tail of AeOBP22 is well ordered even in the absence of ligand (Fig. 3b**)**. Therefore, we propose it functions as a selectivity filter for specific ligands of the appropriate chain length.

Previous studies of AeOBP22^4,59^ showed that the length of the ligand was critical for binding, and also branching of the alkyl chain was detrimental to binding indicative of binding to a narrow pocket, consistent with our findings. However, in these studies the “best” ligands were compounds containing two aromatic rings. Such compounds would only partially occupy the ligand binding pocket we observe, and in agreement with this show binding affinities an order of magnitude weaker than long chain fatty acids. We cannot rule out the possibility that ligands other than fatty acids bind to AeOPB22, and indeed, our initial screens identified small molecules that induce conformational changes but only at much higher concentrations (>1 mM) and in a concentration dependent manner, suggesting that these if these compounds bind *in vivo* they likely require multiple ligands to induce a stable conformation of the protein. Our structural studies provide strong evidence that a negatively charged head group is required on the ligand given the number and arrangement of positively charged residues that surround the ligand binding pocket. Our preliminary screens failed to identify lyso-phosphatidic acid (LPA) (C16-LPA) as a ligand, as it did not produce the same stabilization of AeOBP22 we observed with free fatty acids. We now interpret this as because the overall chain length C16-LPA is too long (23 linear heavy atoms) to be accommodated in the binding pocket and indeed we found that fatty acids longer than C20 chains bind AeOBP22 with dramatically reduced affinities.

Fatty acids components of human sweat and skin and have critical roles in regulating mosquito behaviors. Dodecanoic acid and palmitoleic acid are strong oviposition attractants in *Ae. aegypti*, whilst the saturated C14–C16 and C18 acids are attractants at low concentrations but repellents at high concentrations^85^. In contrast, methyl ester derivatives were found to be oviposition deterrents^85^. Carboxylic acids have also been shown to synergize the effect of lactic acid as an attractant^86^. Carboxylic acids with C1–C3, C5–C8 and C13–C18 alkyl chains acids all increased attraction compared to lactic acid alone, whereas, C4 and C9–C12 had no effect or showed a decrease in attraction. Whilst other studies have suggested that C9 and C10 acids have a higher stimulatory effect compared to other fatty acids^87^. Our structural studies indicate that AeOBP22 retains the ability to bind to short chain carboxylic acids suggesting that AeOBP22 may have the ability to recognize different ligands in different tissues antennal AeOBP22 may modulate chemosensory responses to volatile short chain fatty acids, whether it functions as a transporter or a buffer of ligand concentration remains unknown^20^. Within the insect chemosensory system, fatty acids are endogenous components of the sensillar lymph fluid^18^, and these fatty acids can interact with both pheromones and PBPs on external sites to regulate pheromone accessibility and interactions between the PBP and the pheromone. In our NMR studies of AeOBP22 even at high fatty acid concentrations, we only observe a single binding site for the fatty acid and no evidence of other alternative sites of interaction.

In the proboscis AeOBP22 could be function to recognize fatty acids. However, its function in the salivary gland and male reproductive tissue is less clear. As a secreted protein in the salivary gland^6,7^ it is likely injected into the host during a blood meal. The closely related protein PregOBP56a from the blow-fly *P. regina* has been proposed to sequester and transport lipids for feeding^71^. It is unlikely that AeOBP22 plays such a role in the mosquito given the difference in the mechanisms of feeding. Rather, it seems that it must function to either transport a required ligand from the mosquito to the host or to sequester a signal present in the host. Similarly, given its expression in male reproductive tissue and its transmission to females, it would appear that it must be transporting or sequestering a pheromonal component. Our data suggest that in either case, any compound involved in these processes is likely to be a long chain negatively charged lipid structure, and the ability to undergo a conformational change that enhances binding may be an important evolutionary development that allows it to recognize low abundance compounds with high affinity.

## Materials and Methods

### Protein expression

The mature form of AeOBP22 lacking the N-terminal signal peptide was expressed and purified as previously described^88,40^.

### NMR spectroscopy

NMR experiments were performed at 25 °C on a either a Varian 900 MHz DD2, Varian INOVA 600 MHz or Bruker Avance Neo 600 MHz spectrometer. Samples for NMR were dissolved in sodium phosphate (20 mM, pH 6.5) and 90% H_2_O/10% D_2_O, with DSS (4,4-dimethyl-4-silapentane-1-sulfonic acid) (80 μM) added as an internal reference 89,90. For ligand screening, protein concentration was 100 μM and ligands were added to a concentration of 200–500 μM. For chemical shift assignments, relaxation rate measurements and structure calculations, protein concentrations were in the 400–700 μM range. All NMR experiments were collected using non uniform sampling methods^91^ using the Poisson-gap sampling schemes implemented by Hyberts *et al*.^92^ with a sampling density of 25–50%. Data were processed using the istHMS package v2111^93,94^ in combination with NmrPipe^95^ and analyzed using Ccpnmr Analysis v 2.4.2^96^.

#### Chemical shift assignments

Chemical shift assignments for the complex with arachidonic acid and the backbone assignments for the apo-protein were previously reported^40^. Backbone ^1^H, ^15^N, ^13^Cα and ^13^Cβ assignments for nonadecanoic, linoleic acid (pH 4.5 and pH 6.5), and decanoic acids were made in the same way. All other backbone amide assignments were made by following chemical shift trajectories as a function of ligand concentration or chain length. Normalized chemical shift differences are reported as^97,98^

1 $$\Delta \delta =\sqrt{({\Delta {\rm{\delta }}H}^{2}+0.14\ast {\Delta {\rm{\delta }}N}^{2})}$$

#### pH Titrations

Samples were dissolved in sodium citrate (20 mM, pH 6.5) and the pH adjusted using HCl and measured using a Lazar Ultra-M micro pH electrode (Lazar Labs, Los Angeles, CA).

#### NMR relaxation rates

^15^N R_1_, R_1_ρ and {^1^H}-^15^N heteronuclear NOEs for the apo-protein and the arachidonic acid complex were measured at 900 and 600 MHz with temperature compensation blocks, and the full set of relaxation delays were acquired prior to incrementation of the ^15^N evolution time^99^. Heteronuclear NOEs used a 5 second relaxation delay, with a saturation period of 3 seconds. For R_1_ measurements 11 relaxation delays were acquired spanning 0.01–1.2 seconds at 14 T and 0.01–1.8 s at 21 T. For R1ρ measurements, the spin lock field strength at 21 T was 2250 Hz and at 14 T it was 1829 Hz, and relaxation delays were arrayed over 30–210 ms. R1ρ values were converted into R2 values^100^ prior to analysis using the “d’Auvergne” protocol in the Relax software suite^52,53,101–103^.

#### NMR structure determination

NOE distance restraints were obtained from a simultaneous ^15^N/^13^C separated NOESY-HSQC^104^ with a 100 ms NOE mixing times. Intermolecular NOEs between the protein and ligand were recorded using a simultaneous ^13^C/^15^N F_1_-filtered, F_3_-edited NOESY-HSQC^105,106^ (150 ms mixing time), using samples recorded in both 90% H_2_O/10% D_2_O and a second that was exchanged into 99% D_2_O (pD 6.1). Intermolecular restraints were derived from those NOE correlations that were observed uniquely in the intermolecular NOE spectrum.

Backbone dihedral angle restraints were determined using Talos+^107^. Any dihedral angles that initially appeared over restrained (clustered at the extreme ends of the restraint) were removed from subsequent calculations. Unambiguous side-chain χ1 restraints were identified from ^3^*J*_HAHB_ coupling constants measured as previously described^108^ in combination with NOE measurements. For structure calculations, the Karplus coefficients (A, B, and C)^109^ for ^3^*J*_HAHB_ were parameterized as given in Perez *et al*.^110^.

Backbone amides involved in hydrogen-bonds were identified from an ^1^H-^15^N HSQC spectrum after the sample had been exchanged into 99% D_2_O based buffer. Acceptor groups for the H-bond restraints were identified after structure calculations had converged and these were included in the final iterations of refinement.

Structure calculations were performed using ARIA 2.3.2^111,112^ in combination with CNS-SOLVE (Version 1.2)^113^. NOE restraints lists were generated within CCPNMR analysis and divided into unambiguous and ambiguous restraint sets. Initial iterations used a soft-square well potential for the NOE restraints to identify violations in the restraint lists, and if violated these were subsequently treated as ambiguous restraints. The final rounds of calculations employed the log-harmonic potential in the final cooling stage^114^ in combination with a modified “soft” force field and updated weighting parameters^115^. In the final iteration, a total of 100 structures were determined, and the top 50 were refined in water and the top 30 of these selected for analysis based on their total energy as described^114^. Superposition of structures and calculation of RMSD were performed using the suppose program from Ambertools^116^.

### X-ray crystallography

#### Crystallization

For crystallization trials, AeOBP22 was exchanged into 4-(2-hydroxyethyl)-1-piperazine-ethane-sulfonic acid (HEPES) buffer at pH 7.5. The protein was concentrated to 9–12 mg ml^-1^ and incubated with ligands at room temperature overnight prior to crystallization trials. Crystals were grown by sitting drop vapor-diffusion.

Initial crystals were obtained in the P3_1_21 space group in the presence of benzaldehyde (2 mM), by mixing the protein solution in a 1:1 ratio with a precipitant solution containing sodium-potassium tartrate (0.2 M), sodium citrate (0.1 M) pH 5.0 and ammonium sulfate (2 M). A tantalum bromide derivative was obtained by soaking crystals with a 2 mM Ta_6_Br_12_ solution (Jena Biosciences) for 2–3 days. Crystals of the complexes in the P3_1_ space group were obtained at 18 °C through optimization of an initial hit containing ammonium sulfate (1.2–1.4 M), citric acid (0.1 M), and varying the pH over 5.5–5.7, and optimized by addition of sodium chloride to the well solution across a concentration range of 1.0–1.5 M. Crystals in the C121 space group were obtained using PEG3550 (12% w/v) and HEPES (0.1 M) at pH 7.5. For the C18:2 complex the precipitant solution additionally contained cobalt chloride (4 mM), cadmium chloride (4 mM) and magnesium chloride (4 mM). Arachidonic acid complex crystals were obtained with cobalt chloride (6 mM), cadmium chloride (10 mM) and magnesium chloride (6 mM). The apo-protein crystal was obtained with cobalt chloride (6 mM), cadmium chloride (6 mM) and sodium chloride (6 mM).

#### Data collection

X-ray diffraction data was collected at the Molecular Biology Consortium, Beamline 4.2.2. at the Advanced Light Source, LBNL, Berkley CA (crystal form 1 and 2) and the University of Colorado School of Medicine Biomolecular X-ray Crystallography Center (crystal form 3) using a Rigaku MicroMax™ 007 HF generator with a copper anode and equipped with a Rigaku PILATUS3 R 200 K detector.

#### Structure solution and refinement

The initial structure of AeOBP22 was solved using SAD methods of a Ta_6_Br_12_ soaked crystals in the P3_1_21 space group collected at a wavelength of 1.254 Å. Data were integrated and scaled using XDS^117,118^ and refined to a resolution of 2.59 Å using Phenix^119^ to locate heavy atoms sites and generate initial maps. A native data set collected at 1.0 Å on the same crystal was solved using Phenix^119^ with model rebuilding in Coot^120^ and refined to a resolution of 1.9 Å.

Crystal structures of the complexes in the P3_1_ space group were solved by molecular replacement^121^ using the structure obtain from the Ta_6_Br_12_ crystal as an initial search model. Data were collected at both the ALS beamline 4.2.2 and on the home-source, which was processed with HKL3000^122^. These crystals are pathologically twinned (~46%), and data were refined using intensity based twin refinement in Refmac^123^ within CCP4^124,125^. Structures of the complexes in the C121 space group were solved by cobalt or cadmium SAD methods using data sets collected in house at a wavelength of 1.5418 Å, structures were solved using Phenix and subsequently refined directly against the SAD data in Refmac^123^.

### Ligand binding measurements

#### Affinity of AeOBP22 for 5-(N-dodecanoyl)-aminofluorescein (DAF)

All fluorescence experiments were recorded at 25 °C on a Horiba Fluorolog 3–1–1 spectrofluorometer. DAF spectra were acquired using an excitation wavelength of 490 nm and the emission recorded between 500 and 560 nm at 1 nm intervals with an integration time of 1 s and slit widths of 2 nm. Stock solutions of DAF (Thermo Fisher, Batch D-109) were prepared in methanol with potassium hydroxide (0.2 M) and the concentration measured by UV spectroscopy using an extinction coefficient of 88000 M^−1^ cm^−1^ at 497 nm provided in the certificate of analysis. A 1:100 dilution was made into HEPES (50 mM, pH 8.1) and the sample vortexed and checked by UV to verify the DAF concentration. A solution of DAF (~80–100 nM) was titrated with increasing AeOBP22 to a final concentration of ~4 μM. The fluorescence intensity (cps) at the emission maxima (513 nm) was plotted against the total concentration of AeOBP22 and the *K*_D_ for DAF was determined by fitting the raw fluorescence intensity for each replicate to Eq. (1):

2 $${F}_{i}={F}_{0}(1-([{P}_{T}+{L}_{T}+{K}_{D}]-\sqrt{{[{P}_{T}+{L}_{T}+{K}_{D}]}^{2}-4{P}_{T}{L}_{T}})/2{L}_{T})-{F}_{min}$$

where F_i_ is the measured fluorescence, F_0_ is the initial fluorescence in the absence of any protein, P_T_ is the total protein, L_T_ is the total concentration of DAF, and F_min_ is the residual fluorescence after maximal quenching by the protein.

#### Binding affinity for fatty acids

Binding affinities were determined by monitoring the recovery of DAF florescence as a function of fatty acid concentration. Stock solutions of fatty acid were made by dissolving the fatty acid in 100% DMSO, and then making a 1:1000 dilution into HEPES (50 mM pH 8.1). Samples were vortexed prior to each titration point. The protein/DAF solution contained fixed concentrations of DAF (100 nM) and AeOBP22 (1000 nM). Background controls involved titration of the same DAF solution with solvent or fatty acid which showed no significant effect on the fluorescence intensity of DAF.

As there is a single binding site for the fatty acid and the effect of fluorescence quenching can be completely reversed by increasing concentrations of fatty acids, we concluded that the fatty acid is directly competitive with binding of the DAF reporter. Therefore, the term for *K*_D_ in Eq. 2 can be replaced with Eq. 3.

3 $${K}_{app}={K}_{D}(1+I/{K}_{I})$$

where I is the concentration of the competitor and *K*_I_ is the dissociation constant of the competitor. The emission maxima were fit using Eq. 2 substituted into Eq. 1, with the concentration of the protein and DAF fixed at their known concentrations.

## Supplementary Information

Supplementary Information.

## Data Availability

Coordinates for the AeOBP22 and its complexes have been deposited in the Protein Data Bank (https://www.rcsb.org/) with the accession numbers: 6OTL – TaBr Complex (SAD); 6P2E – TaBr (Native); 6OMW – palmitoleic acid; 6OPB – eicosanoic acid; 6OG0 – apo state; 6OGH – linoleic acid; 6OII – arachidonic acid. 6NBN – NMR structure with arachidonic acid. NMR chemical shift assignments and structural restraints have been deposited with the Biological Magnetic Resonance Bank (http://www.bmrb.wisc.edu/) with accession numbers 27724 and 30550.
