# Supplementary material for: Aedes aegypti Odorant Binding Protein 22 selectively binds fatty acids through a conformational change in its C-terminal tail
Source: Sci Rep. 2020 Feb 24;10:3300. doi: 10.1038/s41598-020-60242-9 (PMC7039890; doi:10.1038/s41598-020-60242-9)
Supplement: Supplementary file 1 — Supplementary Information. [file 41598_2020_60242_MOESM1_ESM.pdf]

Aedes aegypti Odorant Binding Protein 22 selectively binds fatty acids through a conformational change in its C-terminal tail

Jing Wang[1], Emma J. Murphy [1], Jay C. Nix [3] and **David N. M. Jones[1,2] ***

Dept. of Pharmacology (1) and Program in Structural Biology and Biochemistry (2)

University of Colorado School of Medicine, Anschutz Medical Campus,

12801 East 17th Ave, Aurora, CO 80045

and

Molecular Biology Consortium (3), Beamline 4.2.2, Advanced Light Source, Lawrence Berkeley National Laboratory, Berkeley, California, USA

* Contact: david.jones@cuanschutz.edu

Tel. +1 303 724 3600

ORCID DNMJ 0000-0002-7586-3543

Supplementary Table 1: X-ray data collection and refinement statistics

PDB ID	TaBr - SAD 6OTL	TaBr - Native 6P2E	C16:1 6OMW	C20 6OPD	Apo 6OG0	C18:2 6OGH	C20:4 6OII
**Data Collection**							
Space group	$P3_1$ 2 1	$P3_1$ 2 1	$P3_1$	$P3_1$	C 1 2 1	C 1 2 1	C 1 2 1
Cell dimensions	87.76 87.76 59.63 90 90 120	86.93 86.93 58.52 90 90 120	154.21 154.21 58.16 90 90 120	154.26 154.26 58.04 90 90 120	55.78 43.42 48.42 90 91.73 90	55.96 42.84 49.32 90 92.78 90	111.21 40.84 56.28 90 117.24 90
Resolution range (Å)	23.46-20.59 (2.68 - 2.59)	34.89-1.9 (1.97-1.9)	38.12-2.1 (2.18-2.1)	38.1-2.0 (2.07-2.0)	23.84-1.85 (1.92-1.85)	27.95-1.85 (1.92-1.85)	27.78-1.85 (1.91-1.85)
Redundancy	10.7 (9.6)	5.7 (5.4)	3.7 (2.2)	3.4 (2.0)	14.8 (13.4)	15.4 (13.9)	14.6 (13.3)
Completeness (%)	99.4 (96.9)	99.7 (99.9)	97.5 (80.9)	99.9 (99.6)	99.8 (99.1)	99.9 (99.7)	99.4 (99.0)
$I/\sigma(I)$	27.3 (3.2)	25.6 (3.2)	12.4 (2.4)	30.2 (6.4)	53.2 (14.0)	45.4 (28.6)	17.9 (4.2)
CC 1/2	0.983 (0.828)	1.00 (0.889)	0.996 (0.712)	0.999 (0.916)	0.999 (0.991)	0.999 (0.998)	0.998 (0.929)
R-meas	0.095 (0.408)	0.022 (0.352)	0.084 (0.576)	0.048 (0.209)	0.062 (0.249)	0.067 (0.110)	0.114 (0.792)
**Data Refinement**							
Resolution (Å)	2.59	1.9	2.1	2.0	1.85	1.85	1.83
No of reflections	8456 (798)	20419 (2001)	89691 (7315)	102184 (10406)	9895 (954)	10076 (987)	19395 (1900)
R-work	0.209 (0.315)	0.183 (0.248)	0.197 (0.190)	0.176 (0.161)	0.185 (0.206)	0.163 (0.188)	0.188 (0.265)
R-free	0.228 (0.315)	0.200 (0.246)	0.230 (0.235)	0.213 (0.203)	0.229 (0.212)	0.199 (0.285)	0.230 (0.330)
Number of atoms							
Protein	970	1030	8743	8776	974	1024	1991
Ligands	32	31	103	70	4	38	55
Water	6	110	336	165	124	152	177
Overall B-factor	53.55	35.84	30.27	28.87	21.25	21.48	27.66
RMS Deviations							
Bonds	0.009	0.010	0.007	0.007	0.013	0.008	0.008
Angles	1.39	1.31	1.18	1.18	1.62	1.24	1.31
Ramachandran favored/outliers	98.32/0	98.33/0	99.44/0	97.76/0	100/0	100/0	99.58/0
Clash score	3.65	2.90	2.03	3.59	3.17	3.35	2.25

Numbers in parentheses are for the highest resolution shell

Supplementary Table 2: NMR Restraints and Refinement Statistics for AeOBP22-arachidonic acid complex (PDB 6NBN)

	PDB ID 6NBN
**Restraints Used**	
Total NOE Restraints	2293
Sequential (|i-j| = 1)	382
Medium Range (|i-j| <= 4)	362
Long Range (|i-j| >5)	390
Intermolecular	76
Backbone Dihedral Phi/Psi (Talos+)	218
H-bond restraints	44
Side chain $^3J_{HA\text{-}HB}$ restraints	128
**Model Refinement**	
Total No. of Structures	30
Average Pairwise RMSD (res 7-121)	
Backbone (N, CA, C)	0.462 Å
All atoms	1.452 Å
Violations	
NOE (Mean + SD)	0.069 ± 0.041 Å
No of NOE viol > 0.5 Å*	5.04 ±3.38
Max distance violation	0.98
Dihedral (Mean + SD)	0.174 ± 0.051
No of dihedral violation > 5˚	0
Ramachandran (all models)	
% Favored/Allowed/Outliers	95/ 5/ <1
Side Chain Rotamers	
% Allowed/ Outliers	97 /3
Deviations from Ideal Geometry	
Bond lengths (Å)	0.0031 Å
Bond angles (˚)	0.467˚
Impropers (˚)	1.064˚
Ligand (RMSZ)	
Bonds	0.15
Angles	0.71

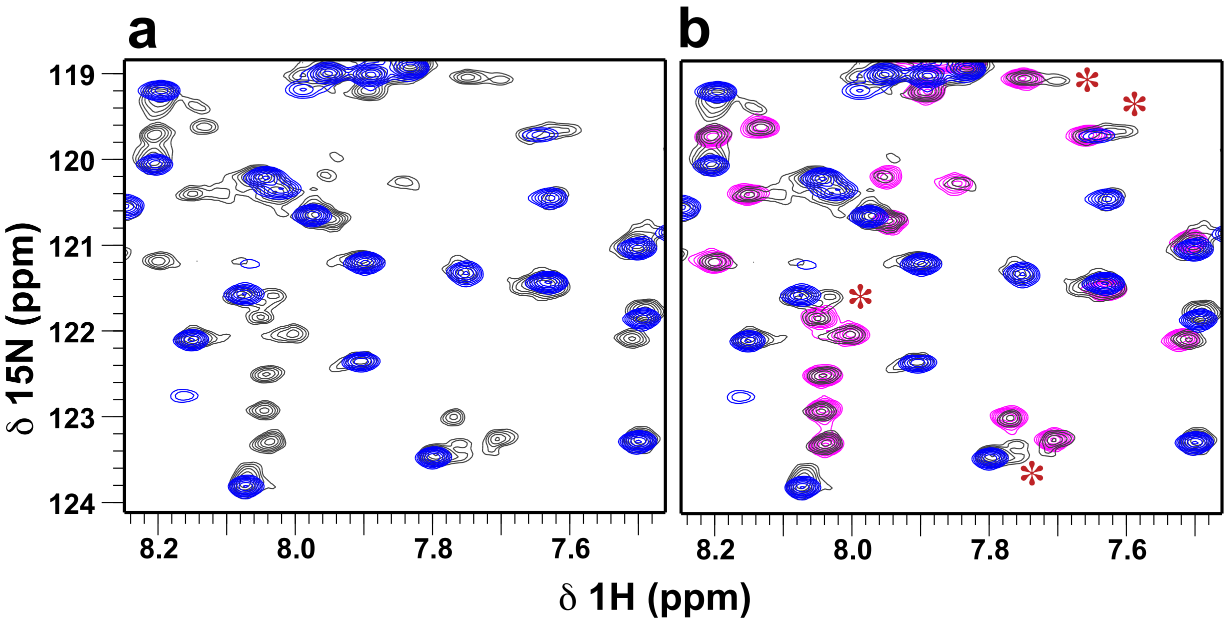

Supplementary Figure 1. Non-delipidated protein is a mixture of the apo protein and complex with palmitic acid (C16). Comparison of a region of the ^{1}H-^{15}N HSQC spectrum of refolded AeOBP22 (black) overlaid with **(a)** the spectrum of the fully delipidated protein (blue) and **(b)** bound to palmitic acid (magenta). The sample consists predominantly of these two species with only minor contributions from some other complex(es) (*) in (b).

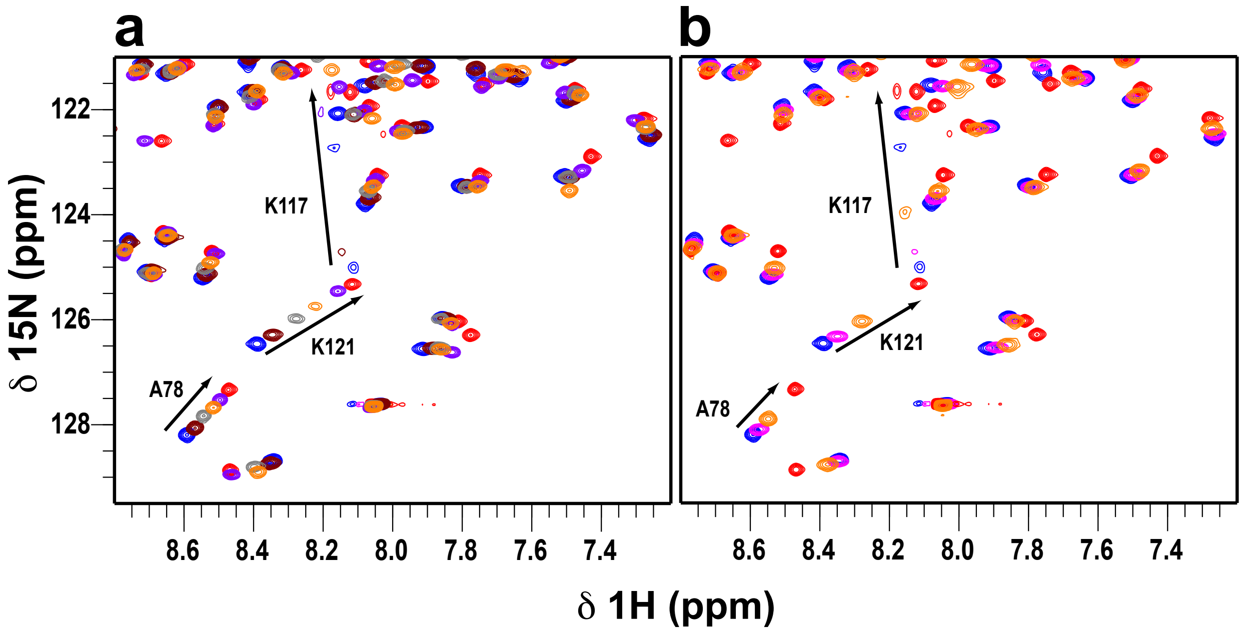

Supplementary Figure 2. NMR based screening for ligand binding to AeOP22.

Region of the ^{1}H-^{15}N HSQC spectrum of AeOBP22 that is highly sensitive to conformational changes in the C-terminal tail showing **(a)** overlay of the apo-state (blue) and in the presence of saturating concentrations of ethyl-butyrate (brown), 6-methyl-5-heptene-2-one (sulcatone, grey),1-octene-3-ol (orange), citronellol (purple) and geraniol (red). **(b)** Changes in the AeOBP22 spectrum (blue), with increasing concentrations of geraniol, 400 μM (magenta), 1 mM (orange) and 5 mM (red). In both cases the protein concentration was 100 μM in sodium phosphate 20 mM, pH 6.5.

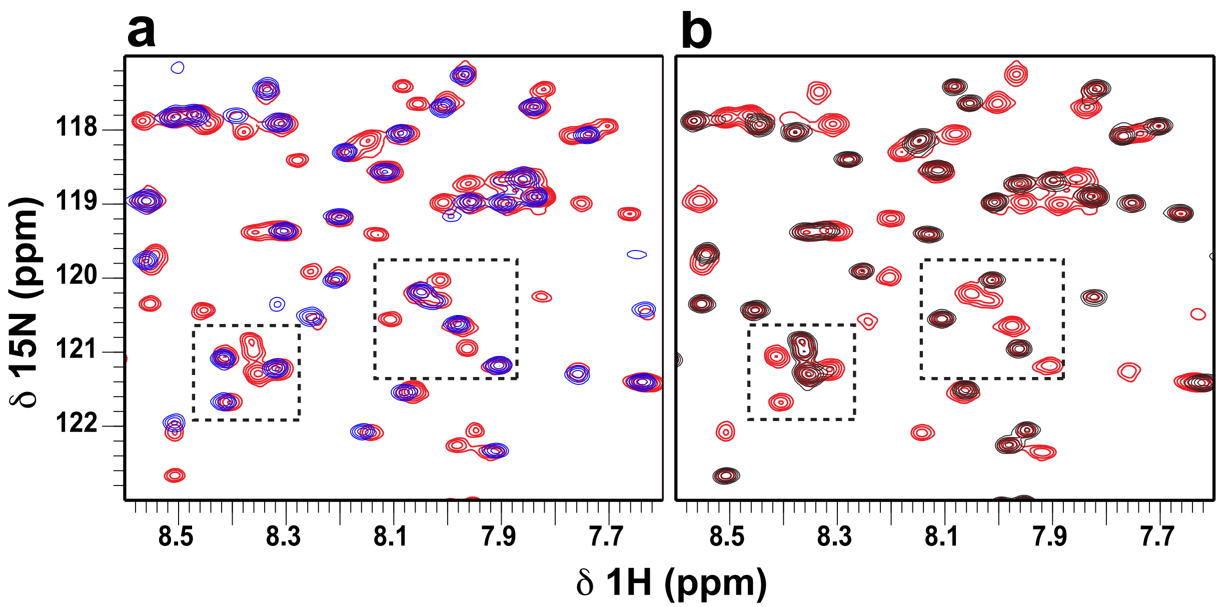

Supplementary Figure 3. Binding of AeOBP22 to long chain fatty acids occurs with high affinity.

Region of the ^{1}H-^{15}N HSQC spectrum of AeOBP22 (100 μM) recorded **(a)** on its own (blue) and in the presence of 0.5 molar equivalents of arachidonic acid (50 μM) (red), showing the presence of both free and bound states **(b)** The same spectra of AeOBP22 complex with 0.5 molar equivalents (red) compared to the spectrum recorded with arachidonic acid in small excess 1:1.1 molar ratio (110 μM) (brown) , which shows the presence of only the bound state of the protein. These spectra indicate that binding occurs with high affinity and the complex is in slow exchange on the NMR timescale.

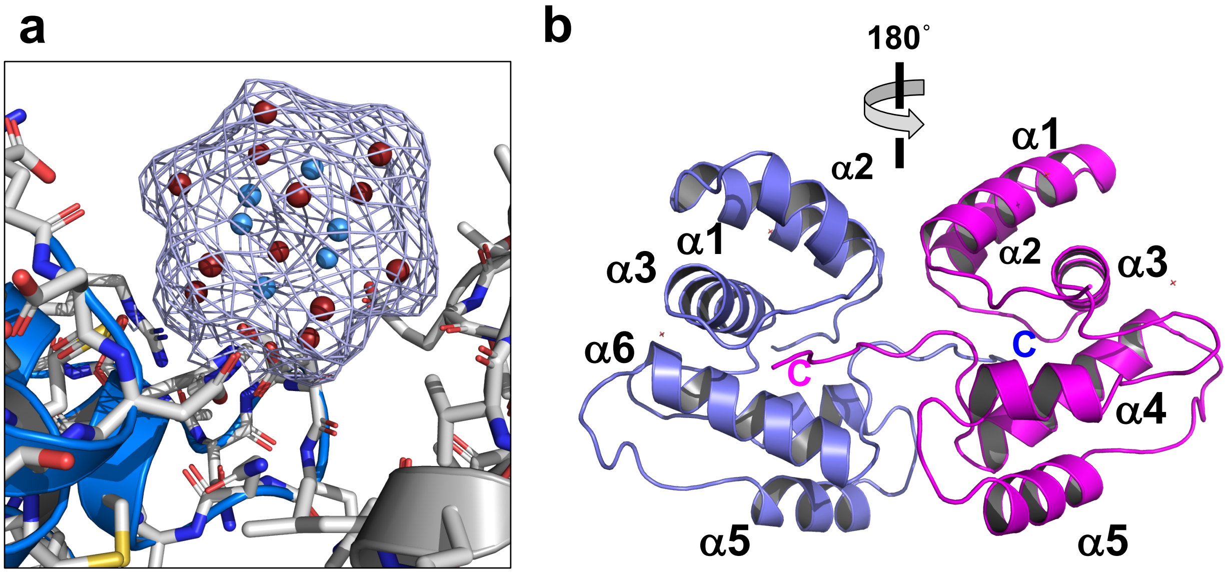

Supplementary Figure 4. Crystal Structure of AeOBP22-Tantalum Bromide Complex.

(a) The electron density of the Ta_6Br_{12} cluster used to solve the structure of AeOBP22 by SAD methods in a 2Fo-Fc omit map of the native data set refined to 1.9 Å. **(b)** Overview of the domain swapped dimer formed in this crystal form in the $P3_121$ space group. The C-terminal residues form an antiparallel beta sheet pairing with a region from the symmetry related molecule.

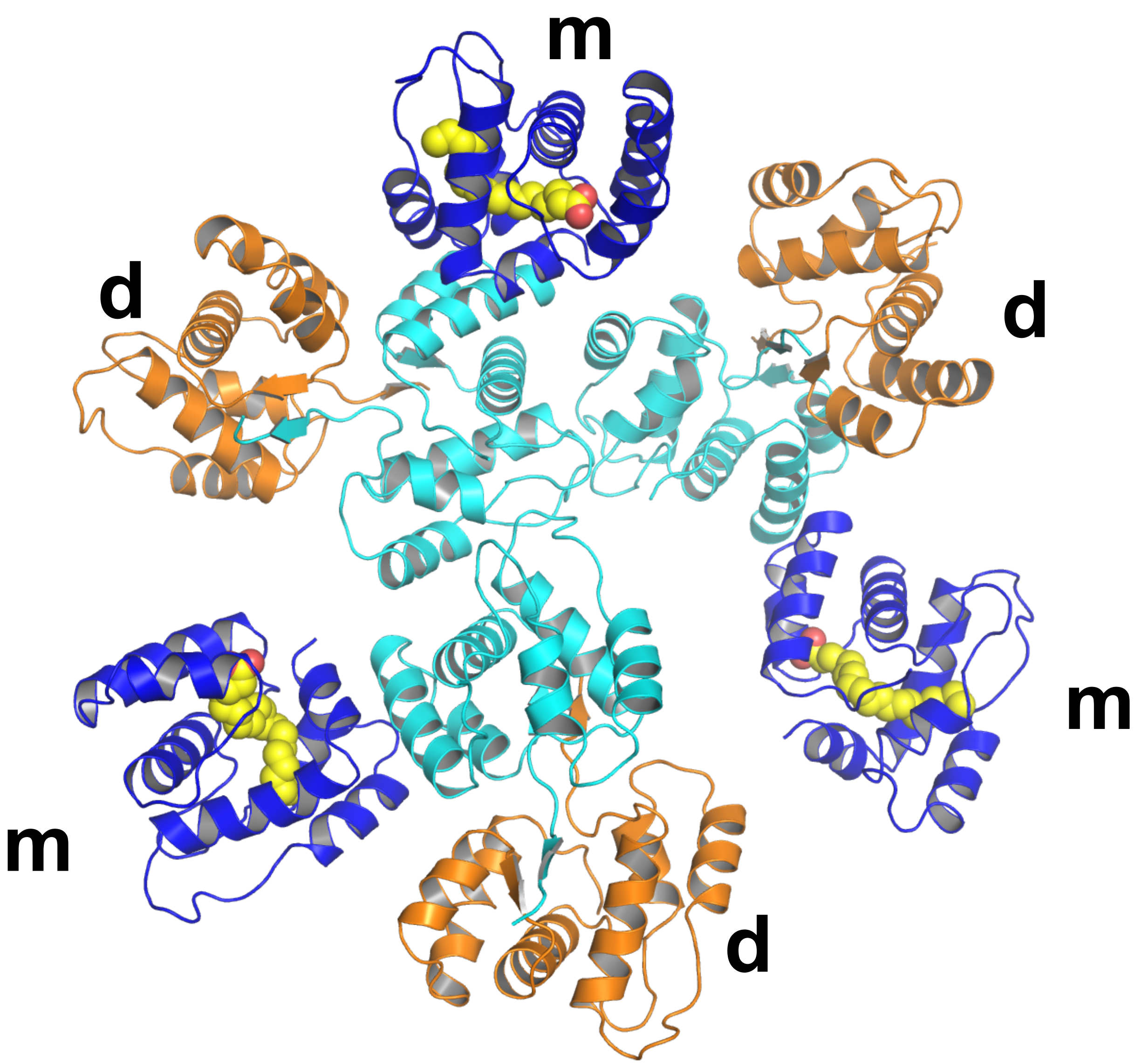

Supplementary Figure 5: Crystal Structure of AeOBP22 in the P3$_1$ space group. This crystal form contains 9 monomers in the asymmetric unit in a pseudo 3-fold arrangement that consists of three swapped dimers (labeled **d** and colored in cyan and orange), each in contact with a monomeric form (labeled **m** and in blue). Each of the monomeric forms of the protein contains a bound fatty acid (shown in space filling representation in yellow). The dimer components do not have any bound ligand.

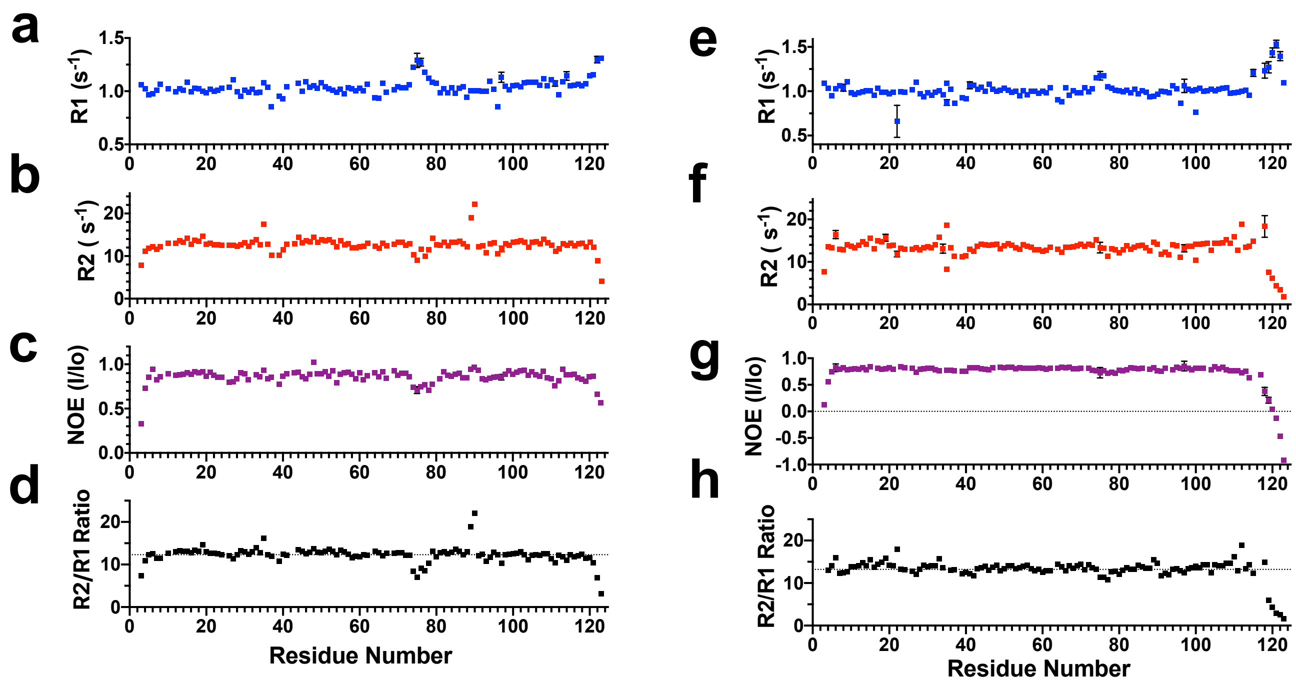

Supplementary Figure 6. Relaxation Data for AeOBP22. Calculated heteronuclear relaxation rates recorded at 900 MHz ^{1}H for the AeOBP22-arachidonic acid complex (**a-d**) and for apo-AeOBP22 (**e-h**). (**a and e)** R_1 for bound and apo forms respectively (**b and f)** R_2**.** **(c and g)** {^{1}H} -^{15}N NOE and **(d and h)** R_2/R_1 ratio. The average value is indicated by the dashed line. For the AA complex this is 12.4 (s.d. 1.7), and for the apo-protein 13.6 (s.d. 1.8). All experiments were recorded in sodium phosphate (20 mM, pH 6.5) at 25˚C as described in [1].

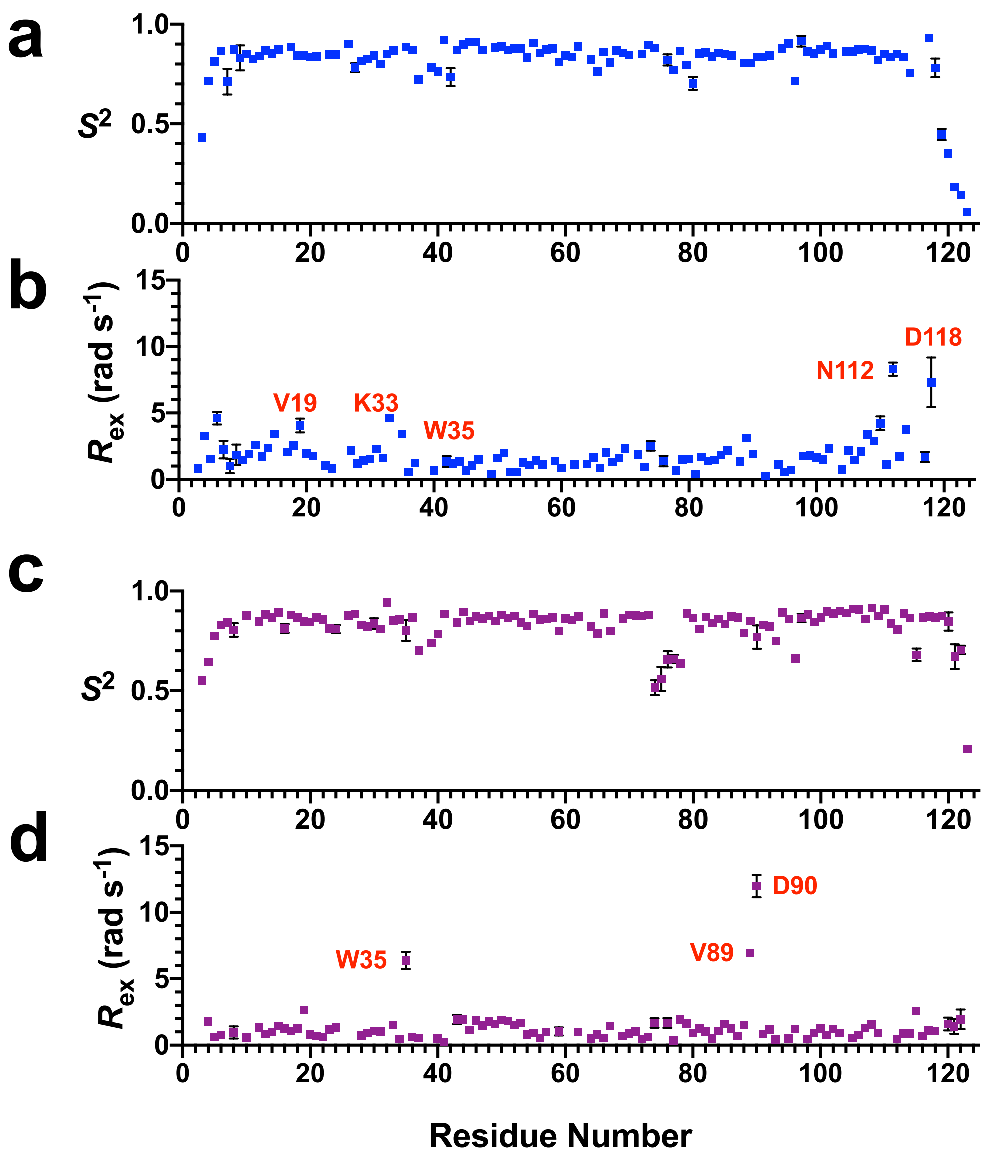

Supplementary Figure 7. Order Parameters and Exchange Contributions to Relaxation Rates. Results of analysis of the relaxation data recorded at both 600 and 900 MHz were analyzed using Relax [2-6] to obtained generalized order parameters (S^2) and R_{ex} for **(a and b)** the apo-protein respectively and **(c and d)** AeOBP22-AA complex. Residues showing enhanced chemical exchange contributions to the R_2 relaxation rates are indicated.

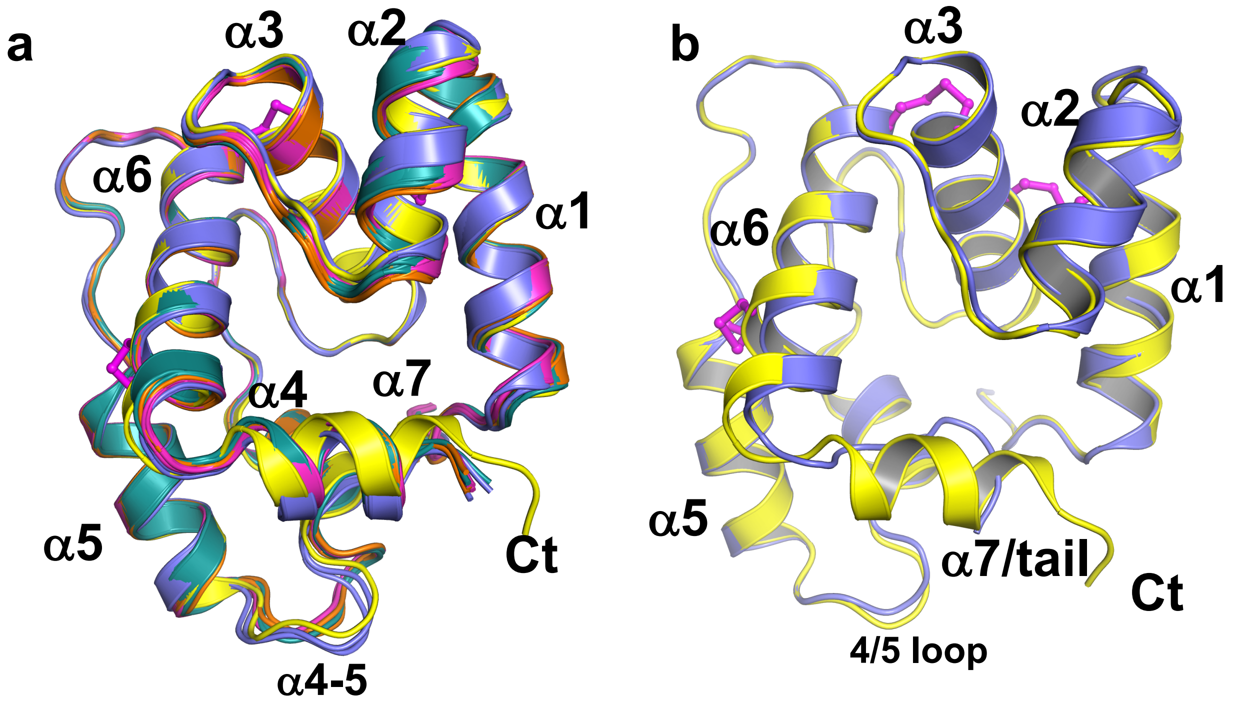

Supplementary Figure 8: Comparison of the monomeric forms of AeOBP22 (a) Superposition of twelve monomers from the different crystal forms of AeOBP22 in the bound states. Structures include the complexes with arachidonic acid (2 structures, shown in blue), linoleic acid (1, yellow), eicosanoic acid (3, orange), palmitoleic acid (3, teal) and palmitic acid (3, magenta). (**b**) Superposition of the linoleic acid complex (yellow) with the structure of the apo-protein (blue) showing the conformational change observed in the C-terminal tail (labeled α7/tail). In both panels the location of the disulphide bridges are shown as magenta sticks.

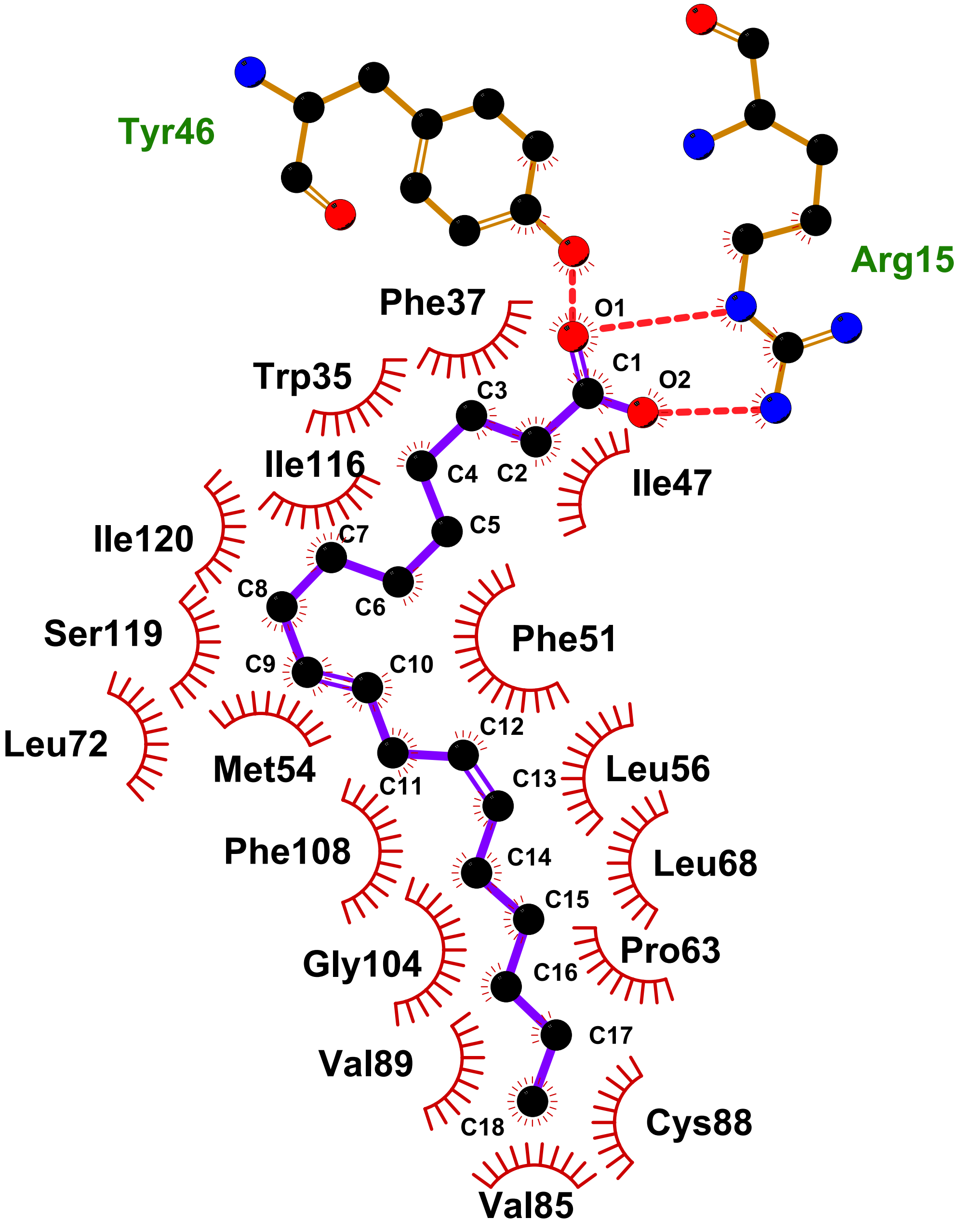

Supplementary Figure 9: Interactions of AeOBP22 with linoleic acid. Representation of the interactions between AeOBP22 and linoleic acid made using LIGPLOT [12]. Tyr46 and Arg15 make hydrogen bonds/electrostatic interactions with the carboxylate head group. The majority of the other interactions are hydrophobic/van der Waals contacts. Note the stereochemistry of the C9-C10 bond is an artifact of the graphical representation.

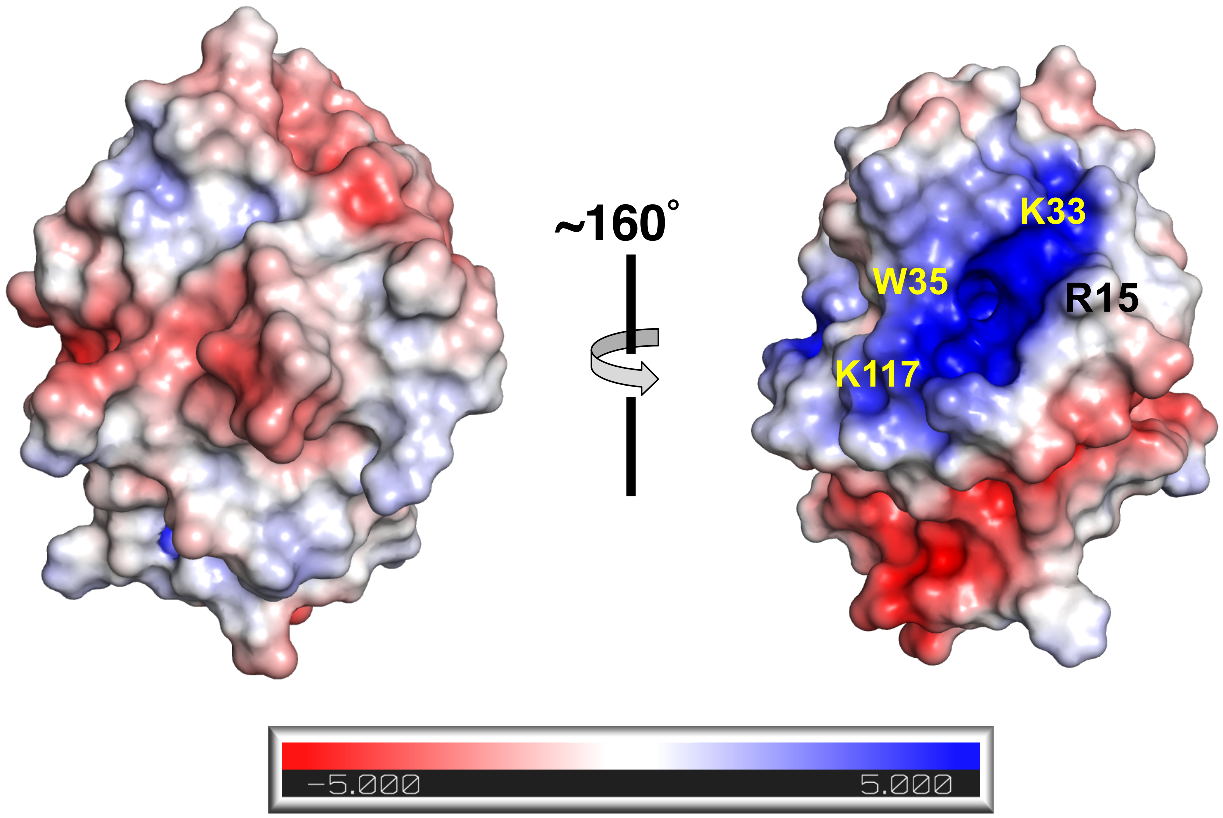

Supplementary Figure 10: Electrostatic Surface Potential of AeOBP22. The electrostatic potential of AeOBP22 mapped to a surface representation of the protein calculated using the APBS tools [7-9] plugin of Pymol [10, 11]. A basic patch formed by R15, K33, K117 and W35 form the entrance to the ligand binding pocket.

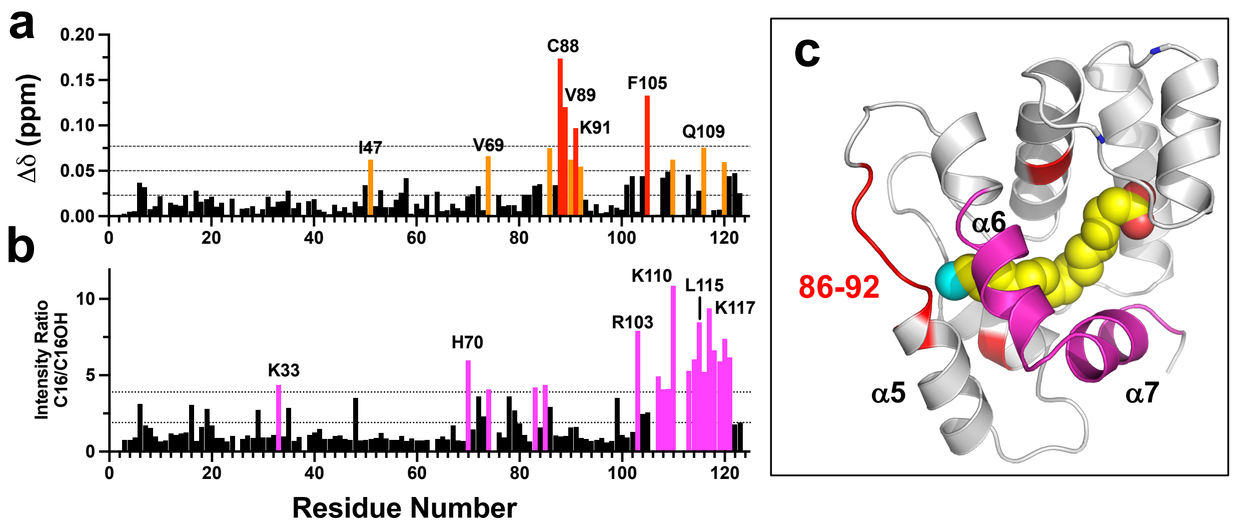

Supplementary Figure 11: Comparison of palmitic acid (C16) and 16-hydroxy-decanoic acid (C16-OH) binding to AeOBP22. Binding of C16-OH produces large chemical shift perturbations (a) and reductions in peak intensity indicative of increased conformational averaging compared to the binding of C16 fatty acid. **(a)** Normalized chemical shift differences for the backbone amides comparing C16 to C16-OH. Significant chemical shift changes are color coded as greater than mean +1 s.d. (orange) or mean +2 s.d. (red). Dashed lines indicate the position of the mean, + 1.sd. and +2 s.d. **(b)** Comparison of relative peak intensities in the ^1H-^{15}N-HSQC spectra of C16 to C16-OH expressed as $I_{C16}/I_{C16\text{-}OH}$, to emphasize the location of the most impacted residues (mean + 1 s.d.) (magenta). In this plot a higher value indicates a reduced intensity in the spectrum of the C16-OH complex. Dashed lines indicate the mean and mean + 1 s.d. **(c)** Mapping of residues that show the largest chemical shift changes (red) and changes in peak intensity (magenta) onto the structure of the AeOPB22-C18:2 complex. The predicted position of the OH group for C16-OH is indicated in cyan.

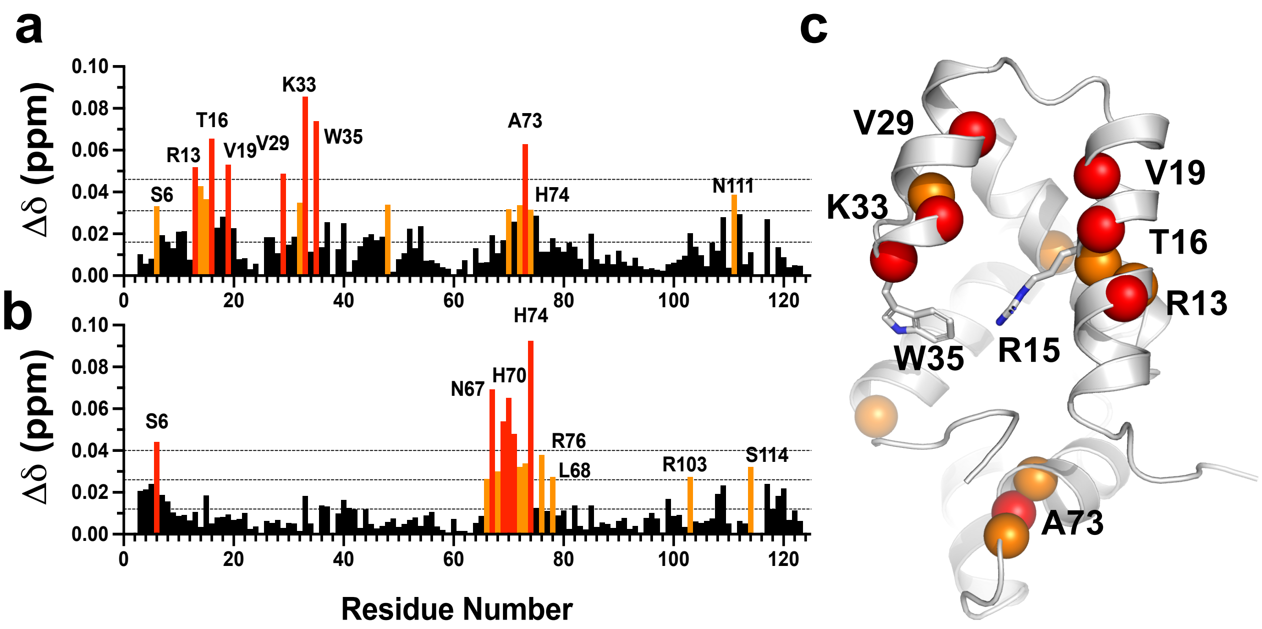

Supplementary Figure 12: Chemical shift perturbations on binding short chain fatty acids. (a) Normalized chemical shift changes in the spectrum of apo-AeOBP22 in the presence of hexanoic acid. (**b**) Normalized chemical shift changes upon changing the pH from pH 6.5 to pH 6.0. Plots are color coded as in Supplementary Fig. 11. **(c)** Backbone amides that exhibit the largest chemical shift changes in (a) mapped onto the apo-structure of the protein cluster in the vicinity of Arg15 and Trp35. Amide groups are represented as spheres and color coded as in (a). The location of R15 and W35 are shown in sticks as reference.

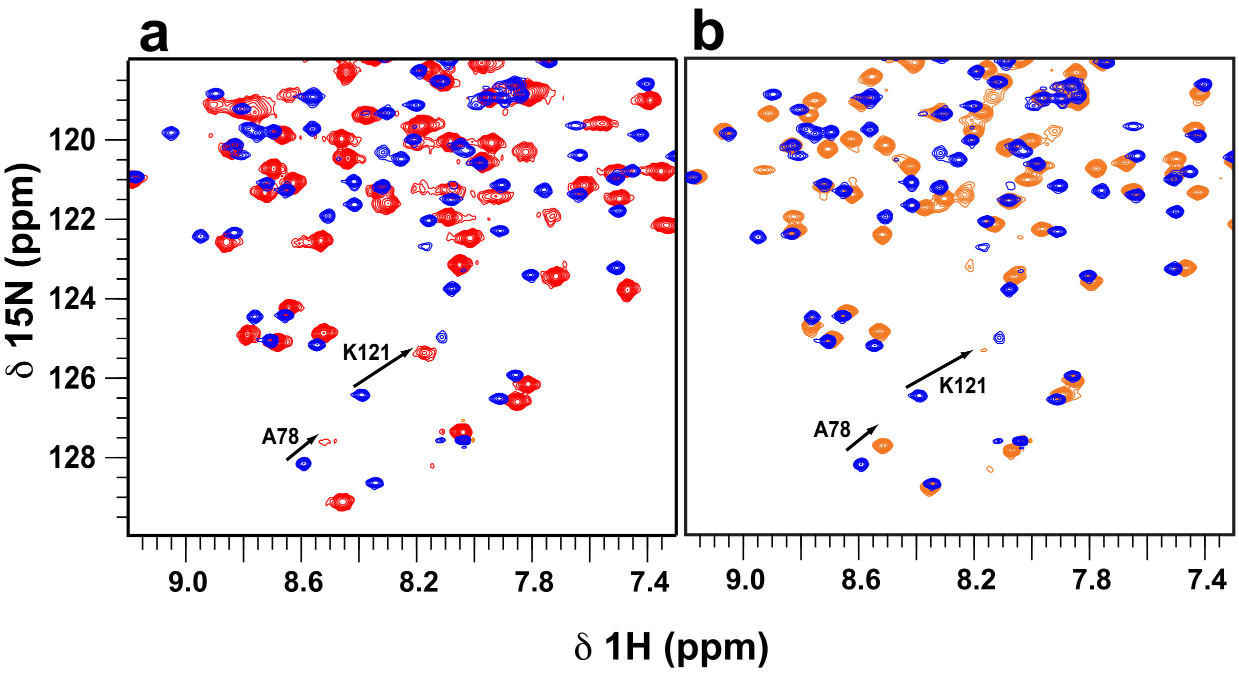

Supplementary Figure 13: Binding of DAF to AeOBP22 induces chemical shifts comparable to other longer chain fatty acids. Comparison of the chemical shift changes that occur in the spectrum of apo-AeOPB22 (blue) in the presence of **(a)** DAF (red) and **(b)** undecanoic acid (C11, orange). Key residues that are highly sensitive to conformational changes in the C-terminal tail are indicated.

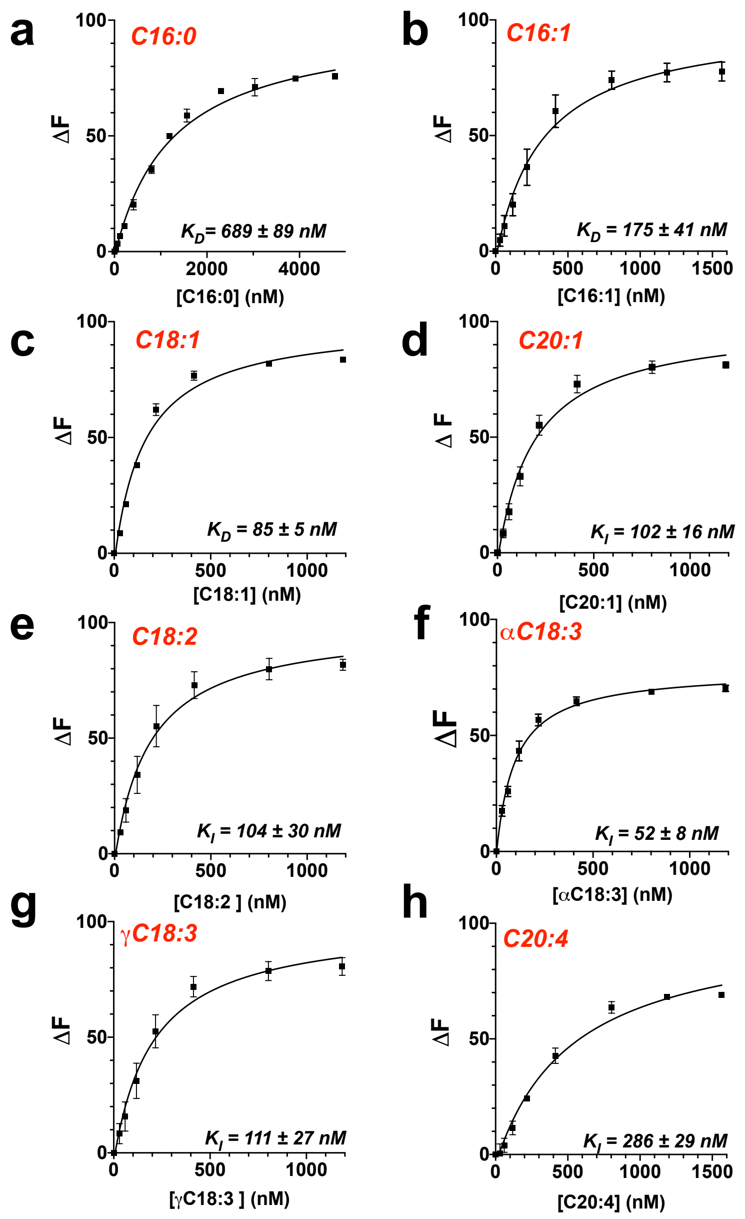

Supplementary Figure 14: Determination of the binding affinity for different fatty acids to AeOBP22. Binding constant determined from the recovery of DAF fluorescence in the presence of increasing fatty acids. The DAF was fixed at 100 nM and the protein was fixed at 1000 nM. Binding constants were determined by fit of the raw data using equations 2 and 3 and a K_D for DAF at 1.194 μM. Results shown for **(a)** Palmitic acid (C16:) **(b)** Palmitoleic acids C16:1, **(c)** Oleic acid C18:1, **(d)** Eicosenoic acid C20:1, **(e)** Linoleic acid C18:2, **(f)** α-Linoleic acid C18:3 (Δ9,12,15), **(g)** γ-Linoleic acid C18:3 (Δ6 ,9,12) **(h)** arachidonic acid C20:4.

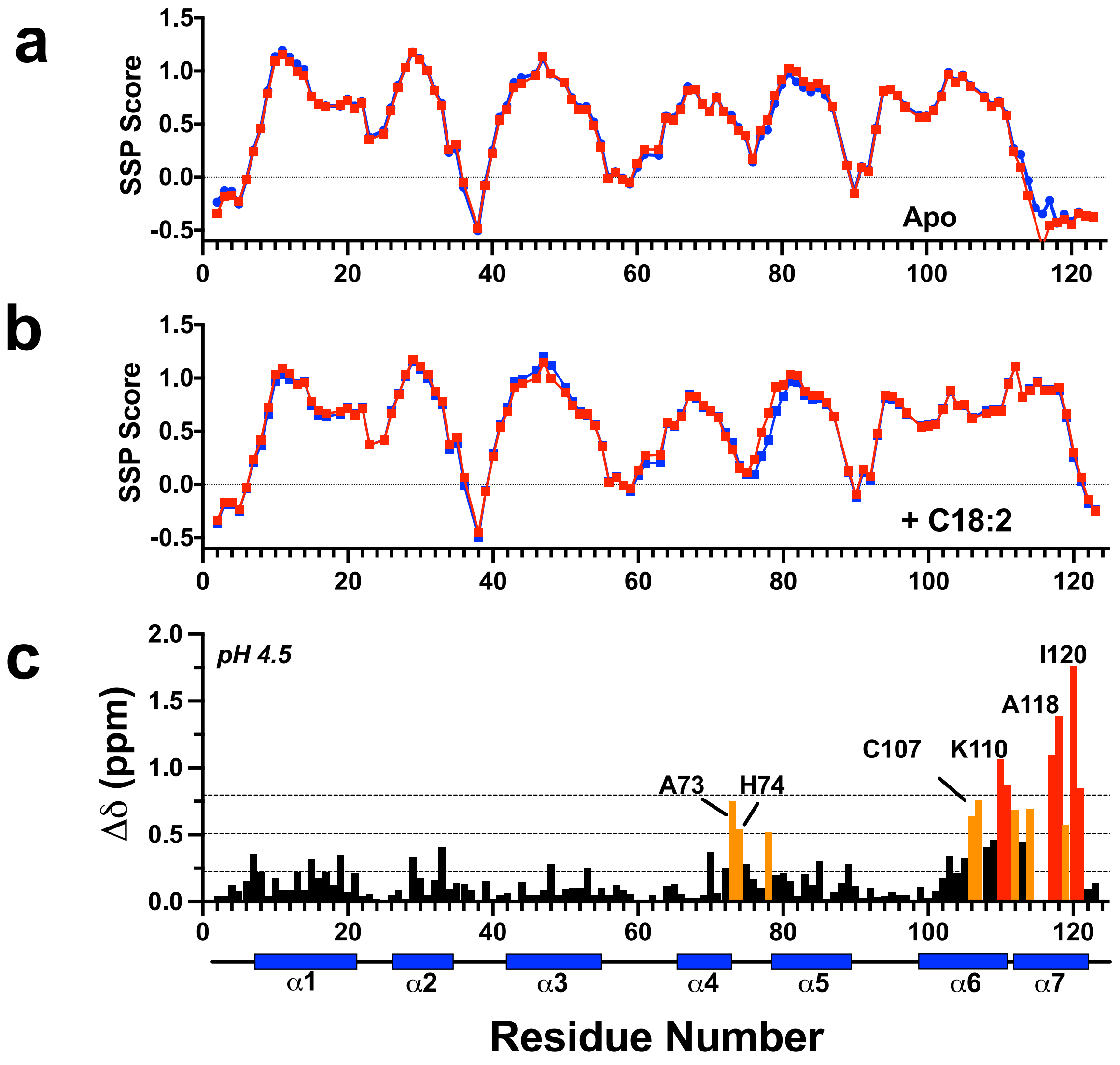

Supplementary Figure 15. Effect of pH changes on the conformation of AeOBP22 Plot of SSP score [13] for **(a)** apo-AeOBP22 at pH 6.5 (blue) and pH 4.5 (red) and **(b)** AeOBP22-AA complex recorded at pH 6.5 (blue) and the AeOBP22-linoleic acid complex recorded at pH 4.5. **(c)** Plot of normalized chemical shift changes for backbone amides between apo AeOBP22 and the complex with α-linoleic acid at pH 4.5. Plot is color coded as in Supplementary Fig. 11. The location of the alpha helical regions is shown as blue cylinders below.

Literature Cited

1. Dayie, K.T. and Wagner, G., "Relaxation-Rate Measurements for N-15-H-1 Groups with Pulsed-Field Gradients and Preservation of Coherence Pathways", *J. Magn. Reson., Ser A*, (1994) **111**: 121-126.
2. Bieri, M., d'Auvergne, E.J., and Gooley, P.R., "relaxGUI: a new software for fast and simple NMR relaxation data analysis and calculation of ps-ns and mus motion of proteins", *J Biomol NMR*, (2011) **50**: 147-55.
3. d'Auvergne, E.J. and Gooley, P.R., "The use of model selection in the model-free analysis of protein dynamics", *J Biomol NMR*, (2003) **25**: 25-39.
4. d'Auvergne, E.J. and Gooley, P.R., "Model-free model elimination: a new step in the model-free dynamic analysis of NMR relaxation data", *J Biomol NMR*, (2006) **35**: 117-35.
5. d'Auvergne, E.J. and Gooley, P.R., "Optimisation of NMR dynamic models II. A new methodology for the dual optimisation of the model-free parameters and the Brownian rotational diffusion tensor", *J Biomol NMR*, (2008) **40**: 121-33.
6. d'Auvergne, E.J. and Gooley, P.R., "Optimisation of NMR dynamic models I. Minimisation algorithms and their performance within the model-free and Brownian rotational diffusion spaces", *J Biomol NMR*, (2008) **40**: 107-19.
7. Baker, N.A., Sept, D., Joseph, S., Holst, M.J., and McCammon, J.A., "Electrostatics of nanosystems: Application to microtubules and the ribosome", *Proc. Natl. Acad. Sci. USA*, (2001) **98**: 10037.
8. Holst, M.J. and Saied, F., "Multigrid solution of the Poisson - Boltzmann equation", *J. Comput. Chem.*, (1993) **14**: 105-113.
9. Holst, M.J. and Saied, F., "Numerical solution of the nonlinear Poisson–Boltzmann equation: Developing more robust and efficient methods", *J. Comput. Chem.*, (1995) **16**: 337-364.
10. DeLano, W.L., *The PyMol Molecular Graphics System*, 2002, DeLano Scientific: San Carlos, CA, USA.
11. Schrodinger, LLC, *The PyMOL Molecular Graphics System, Version 1.8*, 2015.
12. Wallace, A.C., Laskowski, R.A., and Thornton, J.M., "LIGPLOT: a program to generate schematic diagrams of protein-ligand interactions", *Protein Eng*, (1995) **8**: 127-34.
13. Marsh, J.A., Singh, V.K., Jia, Z., and Forman-Kay, J.D., "Sensitivity of secondary structure propensities to sequence differences between alpha- and gamma-synuclein: implications for fibrillation", *Protein Sci.*, (2006) **15**: 2795-804.
